# Super High Capacity of Lithium Battery Silicon–Carbon Anode over 6,500 mAh g^−1^

**DOI:** 10.34133/research.1179

**Published:** 2026-03-13

**Authors:** Shisheng Lin, Minhui Yang, Zhuang Zhao, Mingjia Zhi, Xiaokai Bai, Kangchen Xiong

**Affiliations:** ^1^ College of Information Science and Electronic Engineering, Zhejiang University, Hangzhou 310027, P. R. China.; ^2^ State Key Laboratory of Extreme Photonics and Instrumentation, Zhejiang University, Hangzhou 310027, P. R. China.; ^3^ Zhejiang DiManXi Technology Co., Ltd., Zhejiang, P. R. China.; ^4^ School of Materials Science and Engineering, Zhejiang University, Hangzhou 310027, P. R. China.

## Abstract

As silicon anodes approach their theoretical capacity limits in lithium-ion batteries, the exploration of materials with even higher energy storage potential becomes imperative. Here, we demonstrate that silicon–carbon composites can deliver ultrahigh capacities exceeding 6,500 mAh g^−1^, benefiting from the abundant internal defects within the composite. At a charge–discharge rate of 0.1 C (0.42 A g^−1^), the initial discharge specific capacity reaches 6,694.21 mAh g^−1^, with a Coulombic efficiency (CE) of 74.71%, markedly exceeding the theoretical capacity limit of silicon. By further optimizing lithium battery electrolyte, the initial discharge specific capacity is 5,294.88 mAh g^−1^ and CE is increased to 90.96%. Moreover, an artificial intelligence-assisted framework combining a multilayer perceptron with a constrained genetic algorithm predicts a theoretical maximum initial discharge capacity of 7,789.55 mAh g^−1^. These results provide compelling evidence that silicon–carbon composites hold great promise for substantially enhancing the energy density of next-generation lithium-ion batteries.

## Introduction

Secondary (also called rechargeable) batteries are the most convenient form of energy storage devices. Among all commercially available secondary batteries, lithium-ion batteries offer the highest energy density and therefore have great potential to meet the energy-storage requirements of vehicles, power grid systems, smartphones, and many other consumer electronics [1]. Nevertheless, breakthrough technologies are highly needed for increasing the energy density, where the cathode and anode materials are important [2,3]. Metallic lithium, with a theoretical capacity of 3,860 mAh g^−1^, is often considered the ultimate anode material. However, severe safety concerns and dendrite formation hinder its practical deployment [4–7]. Compared to lithium, silicon-based anodes are attractive due to their high theoretical capacity and low cost. Through alloying reaction with Li^+^, fully lithiated silicon forms Li_15_Si_4_, corresponding to a maximum capacity of 3,580 mAh g^−1^ [8]. Numerous silicon anode architectures have been proposed, including porous silicon, silicon nanowires, silicon nanoparticles, graphene/silicon composites, and amorphous silicon [9–15]. However, the maximum capacity of silicon anode is limited below 3,580 or 4,200 mAh g^−1^ [16]. This limitation raises a fundamental materials question: beyond conventional alloying reactions, are there alternative lithium storage pathways in silicon-based systems that have not been fully explored?

Recent studies suggest that defect engineering provides a promising route to extend lithium storage behavior beyond those in pristine crystalline silicon. Prior studies show that increasing vacancy concentration in Si or other host lattices can raise lithiation rates and provide extra accessible insertion sites and diffusion pathways for Li ions, which boosts single-ion diffusivity and local storage capacity [17]. Studies on twin boundaries or engineered grain boundaries have shown that these extended defects can act as fast diffusion conduits or strain-tolerant pathways to accelerate ionic transport and improve rate performance [18,19].

In silicon–carbon composites, the presence of a conductive carbon matrix further stabilizes defect-rich silicon domains and suppresses catastrophic pulverization. Carbon confinement not only enhances electrical conductivity but also limits silicon crystallite size, promoting partial amorphization and preserving short-range order after repeated lithiation–delithiation cycles [20]. Such defect-stabilized silicon nanodomains may sustain unusual high reversible lithium storage without complete structural collapse, thereby offering a viable materials pathway toward ultrahigh-capacity anodes. In this work, the silicon–carbon composites are fabricated to contain a high density of defects. Beyond increasing the number of available lithiation sites, these defects are proposed to modify the local electronic environment of silicon, facilitating strong Li–Si interactions and promoting correlated lithium transport under nonequilibrium conditions. Experimental results reveal that, at a charge–discharge rate of 0.1 C, the composite delivers an initial discharge specific capacity of 6,694.21 mAh g^−1^ and a Coulombic efficiency (CE) of 74.71%, far exceeding the theoretical capacity limit of silicon. After electrolyte optimization, the initial discharge specific capacity reaches 5,294.88 mAh g^−1^, accompanied by an increase in the CE to 90.96%. This study provides evidence that silicon–carbon anode has the potential to break the theoretical limitations of silicon anode. Such defect-assisted transport may enable transient lithium-rich configurations that go beyond the limitation of conventional storage, providing a plausible explanation for the unusually high specific capacities observed in this study, while further investigations are still required to fully clarify the underlying transport mechanism.

## Results and Discussion

**Fig. 1.**
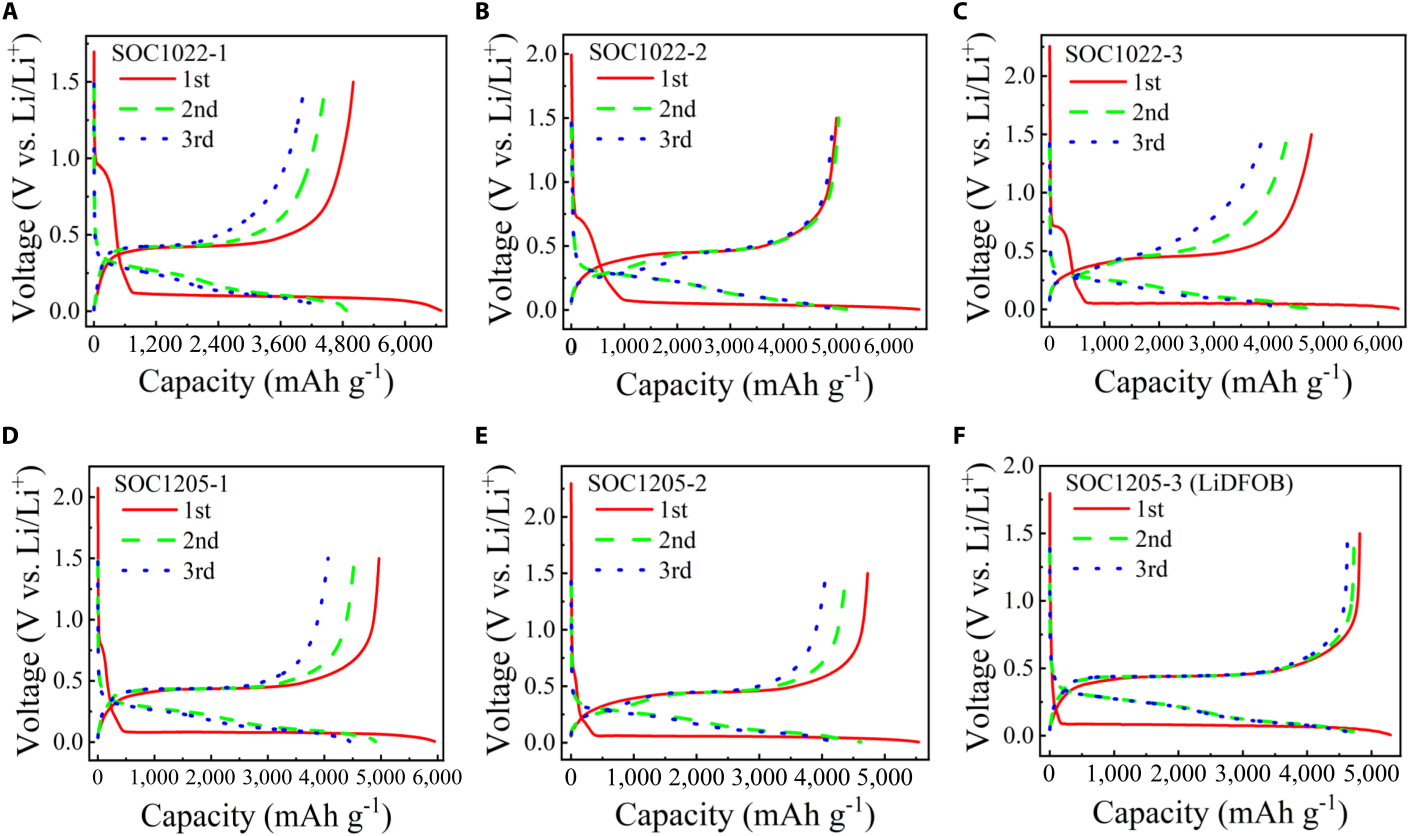
Electrochemical characterization of high-specific-capacity silicon–carbon composite anodes upon cycling. (A) GCD at 0.42 A g^−1^ for the first 3 cycles for sample SOC1022-1. (B) GCD at 0.42 A g^−1^ for the first 3 cycles for sample SOC1022-2. (C) GCD at 0.42 A g^−1^ for the first 3 cycles for sample SOC1022-3. (D) GCD at 0.42 A g^−1^ for the first 3 cycles for sample SOC1205-1. (E) GCD at 0.42 A g^−1^ for the first 3 cycles for sample SOC1205-2. (F) Performance of SOC 1205-3 electrode in 1.0 M LiDFOB in EC:DMC = 1:1 vol% electrolyte.

The electrochemical performance was comprehensively investigated using button-type half-cells assembled with the active material electrodes and metallic lithium counter/reference electrodes, aiming to evaluate their suitability as anode for lithium-ion batteries. Figure 1 presents the galvanostatic charge–discharge (GCD) profiles of the silicon–carbon composite anodes at a rate of 0.1 C for the first 3 cycles, within a voltage window of 0.005 to 1.5 V. The current density corresponding to 1 C is defined as 4,200 mA g^−1^. Three sample series, namely, SOC1022, SOC1205, and SOC1218, were examined, corresponding to carbon contents of 10%, 20%, and 35%, respectively. The electrode consists of silicon–carbon active material, Ketjen black, and polyvinylidene fluoride (PVDF) binder in a mass ratio of 8:1:1. The specific capacity is calculated based on the total mass of the silicon–carbon active material. As shown in Fig. 1A, the SOC1022-1 sample with ~10% carbon content delivers an initial discharge specific capacity of 6,694.21 mAh g^−1^ with a CE of 74.71%. In the subsequent cycles, the discharge specific capacity decreases to 4,867.27 and 4,394.69 mAh g^−1^ for the second and third cycles, accompanied by CEs of 91.86% and 92.68%, respectively. In Fig. 1B, for sample SOC1022-2, the initial discharge specific capacity is 6,561.36 mAh g^−1^ with a CE of 76.23%; the discharge specific capacity in the second cycle is 5,193.35 mAh g^−1^, with a CE of 97.13%, and in the third cycle, the discharge specific capacity is 5,039.32 mAh g^−1^ with a CE of 98.05%, all exceeding the theoretical limitation of silicon. To verify the reproducibility and reliability of the measurements, an additional sample, denoted as SOC1022-3, was evaluated. The initial discharge specific capacity reaches 6,369.96 mAh g^−1^ with a CE of 75.05%, remaining above 6,000 mAh g^−1^. In the subsequent cycles, discharge specific capacities of 4,773.08 and 4,287.80 mAh g^−1^ are obtained in the second and third cycles, respectively, accompanied by CEs of 91.11% and 91.19%.

Increasing the carbon content during sample preparation leads to a noticeable improvement in the CE. As shown in Fig. 1D, the SOC1205-1 sample with a carbon content of ~20% delivers an initial discharge specific capacity of 5,947.54 mAh g^−1^ with a CE of 83.42%. In the subsequent cycles, the discharge specific capacity is 4,898.25 and 4,445.20 mAh g^−1^ in the second and third cycles, accompanied by CEs of 92.45% and 91.44%, respectively. In Fig. 1E, for sample SOC1205-2, the initial discharge specific capacity is 5,542.98 mAh g^−1^ with a CE of 85.25%; the discharge specific capacity in the second cycle is 4,617.55 mAh g^−1^ with a CE of 94.84% and the discharge specific capacity is 4,272.52 mAh g^−1^ with a CE of 95.10% in the third cycle, all exceeding the theoretical limitation of silicon. The sample with capacity over 5,000 mAh g^−1^ can be prepared consistently, and the CE can be optimized through choosing better lithium battery electrolyte. For sample SOC1205-3 (1.0 M LiDFOB in EC:DMC = 1:1 vol%) shown in Fig. 1F, it exhibits a CE of 90.96% for an initial discharge capacity of 5,294.88 mAh g^−1^; the second discharge specific capacity is 4,850.59 mAh g^−1^, with a CE of 97.52%; the third discharge specific capacity is 4,727.15 mAh g^−1^, with a CE of 97.87%. More electrochemical characterizations of electrolyte-optimized samples are shown in Fig. S1, and all of these samples exhibit an initial CE higher than 90%. The results show that the high capacity of the battery is not strongly dependent on the specific electrolyte environment while the CE can be lifted up through electrolyte optimization. Therefore, it is expected that the initial CE and cycling stability can be improved by investigating the special electrolyte and lithium replenishment. It is noted that all those discharge specific capacities even at the third cycle exceed the theoretical limitation of traditional silicon.

**Fig. 2.**
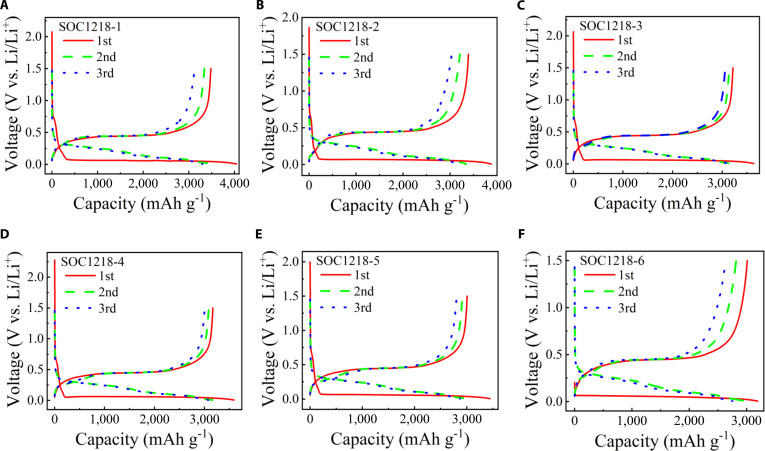
Electrochemical characterization of silicon–carbon composite anodes with high CE. (A) GCD at 0.42 A g^−1^ for the first 3 cycles for sample SOC1218-1. (B) GCD at 0.42 A g^−1^ for the first 3 cycles for sample SOC1218-2. (C) GCD at 0.42 A g^−1^ for the first 3 cycles for sample SOC1218-3. (D) GCD at 0.42 A g^−1^ for the first 3 cycles for sample SOC1218-4. (E) GCD at 0.42 A g^−1^ for the first 3 cycles for sample SOC1218-5. (F) GCD at 0.42 A g^−1^ for the first 3 cycles for sample SOC1218-6.

The CE can be further improved through introducing more carbon atoms in the anode materials. Figure 2 presents the GCD curves of the silicon–carbon composite anodes with a carbon content of ~35%, tested at a 0.1 C rate over the first 3 cycles. The voltage window is set from 0.005 to 1.5 V, with the current density corresponding to 1 C defined as 4,200 mA g^−1^. In Fig. 2A, for sample SOC1218-1, the initial discharge specific capacity is 4,043.01 mAh g^−1^ with a CE of 86.13%; the discharge specific capacity is 3,494.52 mAh g^−1^ with a CE of 95.59%, and 3,295.47 mAh g^−1^ with a CE of 94.88% for the second and third cycle, respectively. In Fig. 2B, for sample SOC1218-2, the initial discharge specific capacity is 3,876.75 mAh g^−1^ with a CE of 87.46%; the discharge specific capacity in the second cycle is 3,342.79 mAh g^−1^ with a CE of 95.95%, and in the third cycle, the discharge specific capacity is 3,148.81 mAh g^−1^ with a CE of 96.37%. This increase in CE is reproducible across multiple samples. In Fig. 2C, for sample SOC1218-3, the initial discharge specific capacity is 3,636.44 mAh g^−1^ with a CE of 88.44%; the discharge specific capacity in the second cycle is 3,213.84 mAh g^−1^ with a CE of 97.73%, and in the third cycle, the discharge specific capacity is 3,128.17 mAh g^−1^ with a CE of 97.84%. In Fig. 2D, for sample SOC1218-4, the initial discharge specific capacity is 3,593.73 mAh g^−1^ with a CE of 88.20%; the discharge specific capacity in the second cycle is 3,161.85 mAh g^−1^ with a CE of 97.65%, and in the third cycle, the discharge specific capacity is 3,076.24 mAh g^−1^ with a CE of 97.75%. In Fig. 2E, for sample SOC1218-5, the initial discharge specific capacity is 3,446.41 mAh g^−1^ with a CE of 87.27%; the discharge specific capacity in the second cycle is 2,995.10 mAh g^−1^ with a CE of 97.30%, and in the third cycle, the discharge specific capacity is 2,872.49 mAh g^−1^ with a CE of 97.70%. In Fig. 2F, for sample SOC1218-6, the initial discharge specific capacity is 3,191.80 mAh g^−1^ with a CE of 94.32%; the discharge specific capacity is 2,970.83 mAh g^−1^ with a CE of 94.78%, and 2,751.63 mAh g^−1^ with a CE of 95.78% for the second and third cycle, respectively. The data presented in Fig. 2 illustrate the charge–discharge behavior of samples exhibiting high initial CEs, ranging from 84% to 95%. Notably, the initial CE reaches as high as 94.32% for the SOC1218-6 sample, corresponding to an initial discharge specific capacity of 3,191.80 mAh g^−1^. The initial discharge specific capacity ranges from 3,000 to 5,000 mAh g^−1^, and can reach up to 4,043.01 mAh g^−1^ (the CE is 86.13%, SOC1218-1). Table 1 shows the initial CE and initial discharge specific capacity of 12 samples in Figs. 1 and 2. The initial discharge specific capacity and CE of samples from SOC1022-4 to SOC1022-7, from SOC1205-4 to SOC1205-17, and from SOC1218-7 to SOC1218-24 are shown in Figs. S2 to S7. For comparison, the capacity of a commercial amorphous silicon–carbon anode material with a nominal capacity of 1,905.5 mAh g^−1^ was measured using our own testing system. The measured capacity of 1,897.42 mAh g^−1^, shown in Fig. S8, further validates the reliability of our experimental measurements.

**Table 1.** The initial discharge specific capacity and Coulombic efficiency of 12 samples in Figs. 1 and 2

Sample name	The initial discharge specific capacity (mAh g^−1^)	Coulombic efficiency (%)	Sample name	The initial discharge specific capacity (mAh g^−1^)	Coulombic efficiency (%)
SOC1022-1	6,694.21	74.71	SOC1218-1	4,043.01	86.13
SOC1022-2	6,561.36	76.23	SOC1218-2	3,876.75	87.46
SOC1022-3	6,369.96	75.05	SOC1218-3	3,636.44	88.44
SOC1205-1	5,947.54	83.42	SOC1218-4	3,593.73	88.20
SOC1205-2	5,542.98	85.25	SOC1218-5	3,446.41	87.27
SOC1205-3	5,294.88	90.96	SOC1218-6	3,191.80	94.32

To further verify the reproducibility of the electrochemical measurements, a statistical analysis was performed on all tested samples within each series. The averaged initial discharge specific capacities of the SOC1022, SOC1205, and SOC1218 groups were 6,480.25 ± 151.37 mAh g^−1^, 5,486.06 ± 284.47 mAh g^−1^, and 4,027.18 ± 420.00 mAh g^−1^, respectively, with corresponding CE values of 71.92% ± 3.70%, 77.80% ± 5.36%, and 85.27% ± 2.67%. These standard deviations confirm the reproducibility of the material synthesis and electrochemical testing processes. The observed trend of higher CE accompanied by a slight decrease in specific capacity demonstrates that rational material design enables the achievement of high initial CE without substantially compromising capacity. Detailed statistical values have been summarized in Table 2, and the weighing data of all samples are shown in Tables S2 and S3.

**Table 2.** The statistical summary alongside the selected examples

Sample group	*n*	Mean capacity (mAh g^−1^)	SD	Mean CE (%)	SD
SOC1022	7	6,480.25	151.37	71.92	3.70
SOC1205	17	5,486.06	284.47	77.80	5.36
SOC1218	24	4,027.18	420.00	85.27	2.67

The *dQ*/*dV* curves for different SOC materials corresponding to the distinct carbon contents are discussed for SOC1205 (lower carbon, ~20%, Fig. S9) and SOC1218 (higher carbon, ~35%, Fig. S10). Both samples exhibit characteristic lithiation/delithiation signatures of silicon-based anodes. The SOC1205 sample displays a sharp cathodic (lithiation) peak at ~0.051 V and a sharp anodic (delithiation) peak at ~0.44 V. The SOC1218 sample, which exhibits a higher initial CE (~85%) compared to SOC1205 (~77%), shows a sharp cathodic peak at ~0.063 V and a sharp anodic peak at ~0.44 V. The consistent anodic peak position across both samples indicates that the fundamental delithiation mechanism remains stable regardless of the carbon content. However, the slight positive shift in the cathodic peak for SOC1218 (~0.063 V) compared to SOC1205 suggests improved lithiation kinetics and reduced polarization in the carbon-rich environment. The sharpness of the peaks in the high-carbon sample (SOC1218) confirms the high reversibility of the redox reactions. This supports our conclusion that increasing the carbon content (carbon alloying) optimizes the CE by establishing a more robust conductive network that buffers volume expansion and stabilizes the electrode/electrolyte interface, thereby minimizing irreversible capacity loss.

**Fig. 3.**
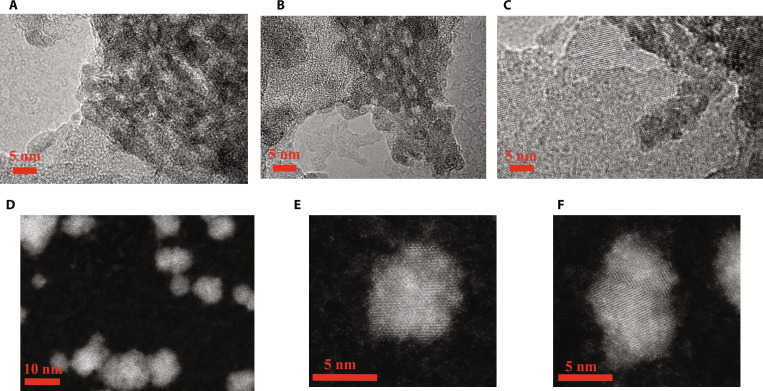
Morphological characterization before cycling. TEM image of graphene-supported Si nanodots (A and B), HRTEM image of graphene-supported Si (C), and HAADF-STEM images of Si nanodots (D to F), where many defects and crystal boundaries can be clearly seen from those images.

Figure 3 shows the TEM images of the silicon–carbon electrode before cycling. As shown in Fig. 3A and B, many silicon nanodots are supported by the graphene sheet, demonstrating the well-defined interface between silicon and graphene by the chemical vapor deposition (CVD) method. From Fig. 3C, the graphene and defective silicon lattice can be clearly seen. Furthermore, we have checked the detailed morphology and lattice of the silicon nanodots by the scanning transmission electron microscopy (STEM) method, where the diameter of the silicon is widely distributed as seen from Fig. 3D. From Fig. 3E, many crystal defects and even noncrystal defects can be observed in the silicon nanodots. From Fig. 3F, the twin boundary can be clearly checked through the high-angle annular dark-field scanning transmission electron microscopy (HAADF-STEM) image, which is in accordance with the fast lithium transport processes inside the anode, leading to the super high capacity of the anodes. Additional PL experiment results can be found in Fig. S11. Under 360 nm excitation, the PL spectra reveal a marked variation in the density of intrinsic defects among the samples. Sample SOC-1022 exhibits a prominent broad emission band centered at approximately 590 nm. In the absence of external doping, this deep-level emission is attributed to the radiative recombination of intrinsic point defects, such as silicon vacancies and vacancy-related complexes. The high intensity of this peak in SOC-1022 indicates a substantial concentration of these radiative defect centers within the crystal lattice. Conversely, the fluorescence is almost entirely quenched in commercial Si/C anode (purchased from Shenzhen BTR Group with a named capacity of 1,000 mAh g^−1^). This dramatic decrease in emission intensity suggests a markedly lower density of deep-level defects in commercial Si/C anode.

**Fig. 4.**
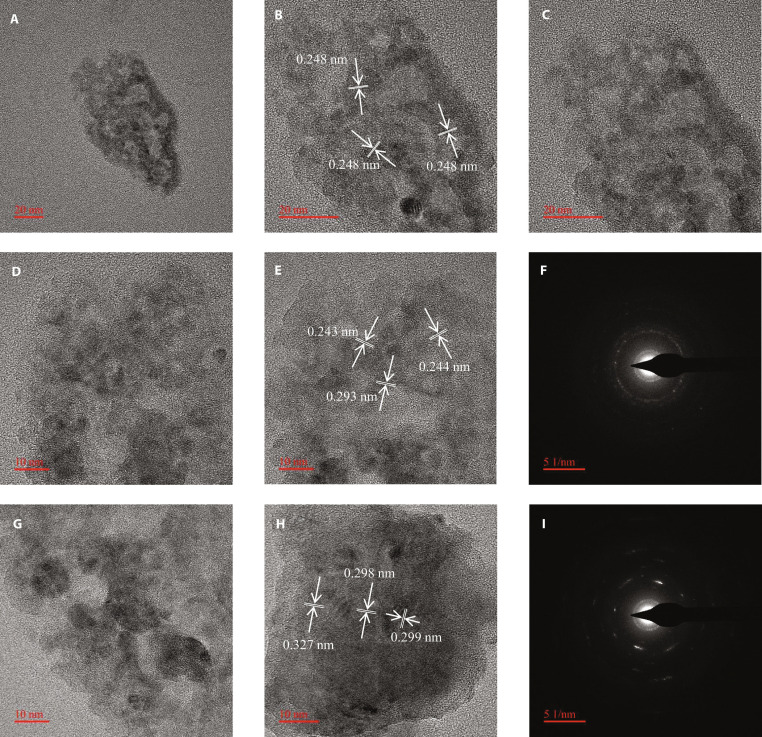
Morphological characterization after 100 cycles. TEM images of the silicon–carbon composite (A to E), and the corresponding SAED pattern of (E) shown in (F). Additional TEM images are presented in (G) and (H), with the corresponding SAED pattern displayed in (I).

Figure 4 shows the TEM images of the silicon–carbon electrode after 100 cycles. Figure 4A to E display the TEM images of the silicon–carbon anode after cycling, while Fig. 4F presents the corresponding selected-area electron diffraction (SAED) pattern of Fig. 4E. Figure 4G and H further show additional high-resolution TEM images of the cycled samples, and Fig. 4I corresponds to the SAED pattern of Fig. 4H. As shown in these images, distinct silicon lattice fringes can still be clearly observed after electrochemical cycling, indicating that crystalline Si domains remain partially preserved within the composite structure. However, the Si lattice exhibits noticeable expansion compared with its standard interplanar spacing. The standard interplanar spacings of crystalline Si are 0.136 nm for the (100) plane, 0.192 nm for the (110) plane, and 0.235 nm for the (111) plane. In contrast, the measured lattice spacings increase to 0.248 nm in Fig. 4B, range from 0.243 to 0.293 nm in Fig. 4E, and range from 0.298 to 0.327 nm in Fig. 4H, respectively. This lattice expansion indicates substantial strain induced by repeated lithiation and delithiation, while the remaining lattice fringes demonstrate partial structural reversibility of the defective Si phase within the silicon–carbon composite.

To further evaluate the structural integrity of the silicon–carbon electrode, additional scanning electron microscopy (SEM) characterization was conducted and is presented in Fig. S12. The SEM images show that the electrode morphology remains largely intact after cycling, with the electrode thickness preserved at approximately 40 μm before and after electrochemical testing, indicating the absence of severe volume expansion or delamination. No obvious particle pulverization or fracture is observed, suggesting that the carbon matrix effectively buffers the internal strain associated with silicon lattice expansion. A slight increase in porosity is detected in the cycled electrode, which may provide accommodation space for the repeated expansion and contraction of silicon domains, thereby mitigating macroscopic cracking. Together with the TEM observations of preserved lattice fringes under strain, these SEM results indicate that the silicon–carbon composite maintains adequate mechanical robustness at the electrode level during lithiation–delithiation processes.

**Fig. 5.**
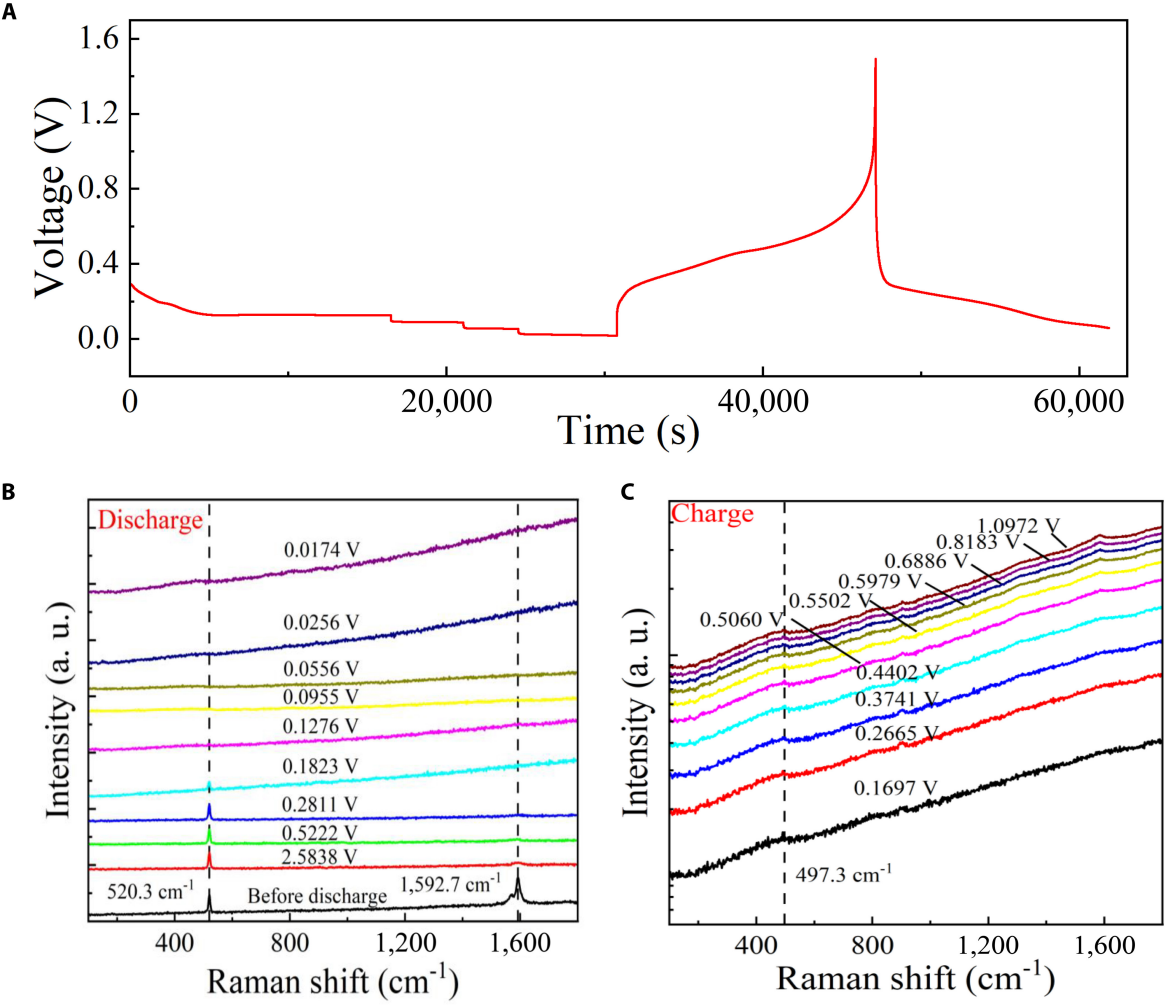
In situ Raman spectroscopy of silicon–carbon composites. (A) The voltage–time curve during discharge–charge cycles. (B) The corresponding in situ Raman spectra collected during discharge. (C) The corresponding in situ Raman spectra collected during charge.

Figure 5 presents the electrochemical and spectroscopic characterization of the Si–C anode during the discharge–charge process. Figure 5A shows the voltage–time profile over one full cycle, displaying characteristic potential plateaus associated with lithium insertion and extraction within the Si–C composite. Figure 5B and C display the in situ Raman spectra collected during discharge and charge, respectively. Before discharge, 2 prominent Raman peaks are observed at 520.26 and 1,592.69 cm^−1^, corresponding to the Si–Si vibration mode of crystalline silicon and the G band of graphitic carbon [21,22]. As the discharge proceeds, both peaks gradually weaken, with the carbon-related G band disappearing earlier than the silicon peak, indicating that the carbon matrix undergoes faster structural or electronic modification during lithiation. When the discharge is completed, both peaks vanish, signifying the substantial disruption of crystalline order as lithium ions insert into the Si lattice [23,24]. Additionally, no obvious electrolyte decomposition was observed during the entire discharge process, and we also performed blank cell control experiments to exclude the contribution of the current collector as shown in Fig. S13. Upon subsequent charging, a new and broad peak appears at 497.30 cm^−1^, which is attributed to the formation of silicon quantum dots with sizes around 2 nm [25,26]. The dissociation of defective silicon into silicon quantum dots and the appearance of peak at 497.30 cm^−1^ is in accordance with Figs. 3 and 4, where the defect promotes the dissociation process. The emergence of this feature suggests that our silicon–carbon composite during lithiation is not fully irreversible; instead, partial recrystallization occurs, forming nanoscale Si domains confined within the carbon framework. These quantum-confined Si regions retain short-range order and indicate that the Si lattice expands but is not completely destroyed. Samples with higher defect densities tend to preserve nanoscale Si domains after cycling.

**Fig. 6.**
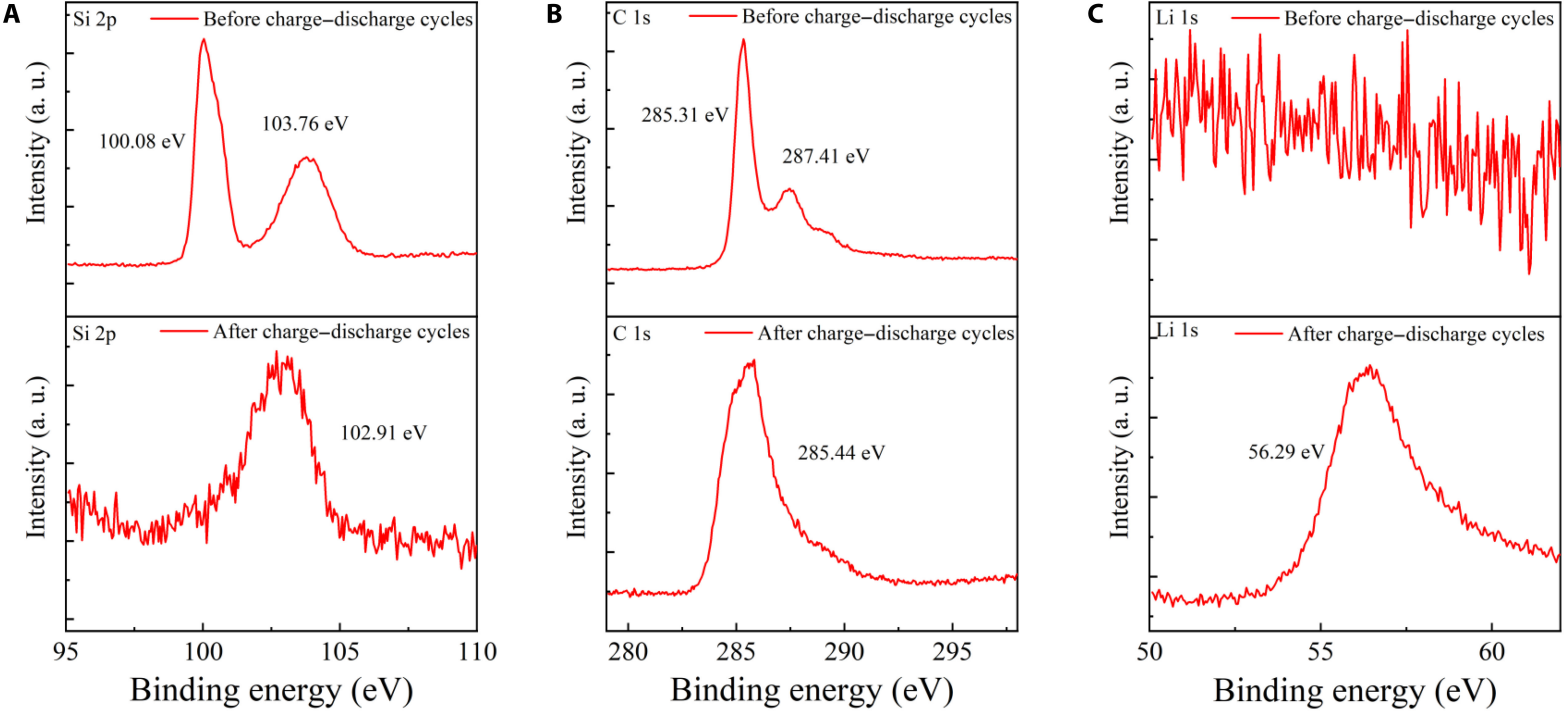
XPS analysis of the silicon–carbon composite before and after cycling. (A) Si 2p spectrum of the silicon–carbon composite. (B) C 1s spectrum of the silicon–carbon composite. (C) Li 1s spectrum of the silicon–carbon composite.

Figure 6 shows the (x-ray photoelectron spectroscopy) XPS spectra of the silicon–carbon composite before and after cycling, including Si 2p (Fig. 6A), C 1s (Fig. 6B), and Li 1s (Fig. 6C) regions. In the Si 2p spectrum (Fig. 6A), 2 distinct peaks are observed at 100.08 and 103.76 eV before cycling, which are assigned to Si–Si bonds of elemental Si and Si–O bonds of surface oxides, respectively, with the Si–Si component being dominant [27]. After cycling, these 2 peaks merge into a single broadened peak centered at 102.91 eV, which can be attributed to the formation of Si–Li bonds, together with a small fraction of sub-oxide or partially oxidized silicon species generated during lithiation and electrolyte decomposition [28]. In the C 1s spectrum (Fig. 6B), 2 peaks are observed at 285.31 eV (C–C/C=C) and 287.41 eV (C=O) before cycling. After cycling, these components merge into a single broadened peak centered at 285.44 eV, indicating the consumption of oxygen-containing functional groups and the formation of a more homogeneous carbon environment. This evolution suggests that the initial surface oxygen species are reduced or incorporated into the solid electrolyte interface (SEI) during lithiation, leaving a dominant C–C bonding character with partial contributions from Li–C species. The Li 1s spectrum (Fig. 6C) shows no signal before cycling but a pronounced peak appears at 56.29 eV after cycling, indicating presence of Li-containing compounds such as LiC_6_, LiF, and Li–Si on the electrode surface [29]. The O 1s spectrum and F 1s spectrum are shown in Fig. S14A and B, respectively. Collectively, these XPS results reveal notable chemical evolution at the Si–C interface upon lithiation. Lithium-ion insertion into the Si lattice causes lattice expansion, while concurrent electrolyte decomposition produces Li-containing compounds that contribute to a stable SEI. The Li 1s signal indicates that lithium exists in a chemically bonded state within the silicon matrix (Li_x_Si) rather than as metallic lithium [30–32]. These findings are consistent with TEM observations of expanded Si lattice fringes and confirm that the silicon–carbon composite maintains structural integrity while undergoing electrochemical and interfacial reconstruction during cycling.

**Fig. 7.**
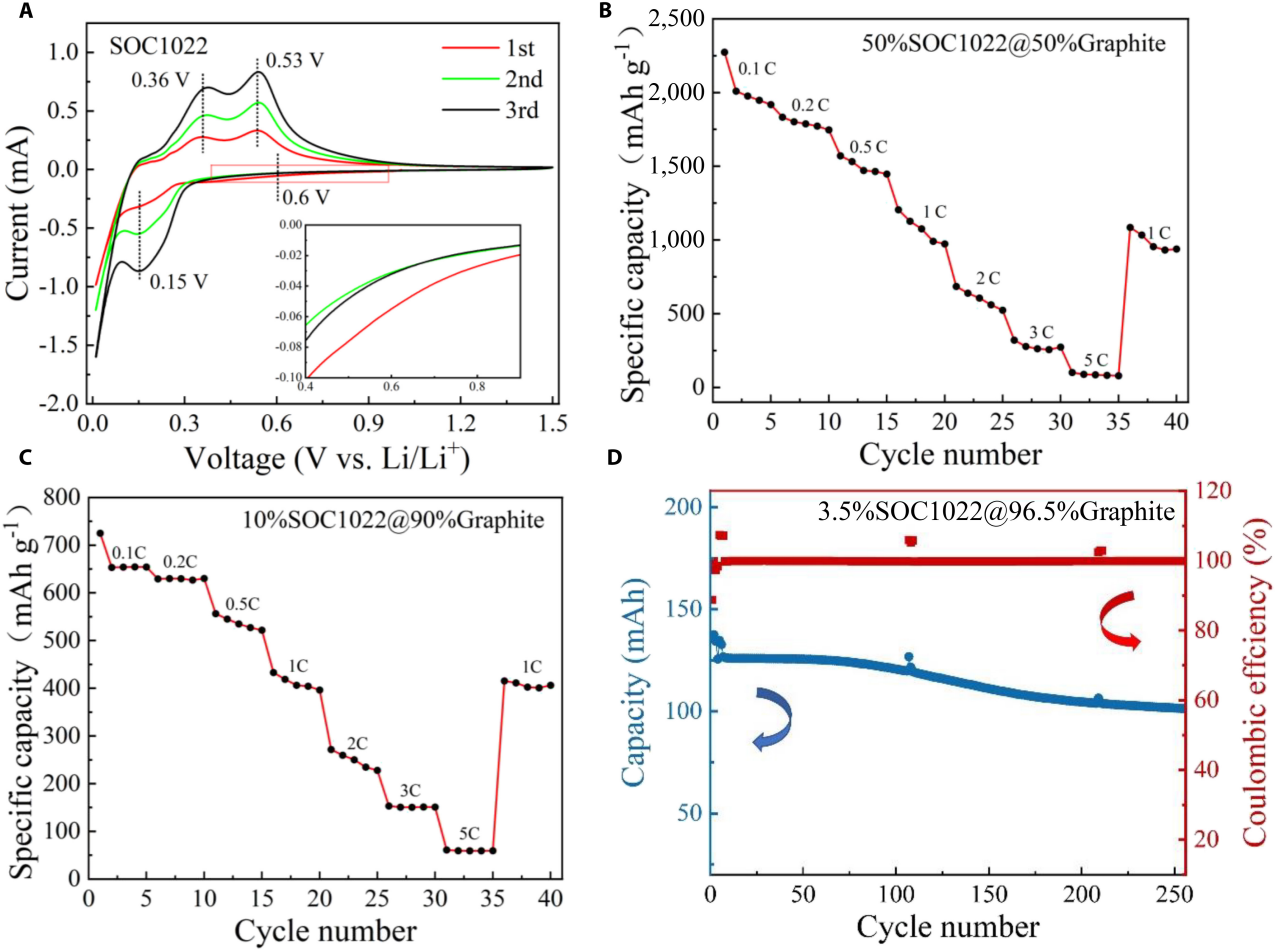
Electrochemical performance of the silicon–carbon composite. (A) CV curves of the SOC1022 sample at 0.5 mV s^−1^. (B) Rate capabilities of electrodes containing 50%SOC1022@50%Graphite sample. (C) Rate capabilities of electrodes containing 10%SOC1022@90%Graphite sample. (D) Cycling performance of the 3.5%SOC1022@96.5%Graphite sample in a pouch cell device.

Figure 7A displays the first 3 cycles of cyclic voltammetry (CV) testing of SOC1022, and the scan rate was 0.5 mV s^−1^. In the first cycle, a broad and weak cathodic peak appeared at approximately 0.6 V, which can be assigned to the formation of the SEI layer [33]. The subsequent cathodic peak at 0.15 V is attributed to the formation of Li silicide (lithiation), which is in line with the 2 anodic peaks at 0.36 and 0.53 V for the delithiation process [34]. In the following cycles, while the peaks corresponding to the lithiation and delithiation processes can still be clearly identified, the peak for the SEI layer disappeared. It should be noted that the current density increases with the number of scan cycles. This is due to the gradual activation of the electrode, which is consistent with previous literature [35,36]. The capacities of 50%SOC1022@50%Graphite and 10%SOC1022@90%Graphite samples at various rates (Fig. 7B and C) further demonstrate the superb rate capability of the silicon–carbon composite materials. To validate practical applicability, we assembled pouch-type full cells by pairing our anode (3.5%SOC1022@96.5%Graphite) with NCM811 cathode to assess their performance. The electrode loading was adjusted to maintain an N/P ratio of 1.1. After electrode stacking and encapsulation in aluminum-laminated pouches, electrolyte was injected, and the cells were vacuum heat-sealed to complete fabrication. The cells were cycled within a voltage window of 2.5–4.2 V. The constant current used for cycling was 140 mA g^−1^ (approximately 0.8 C, based on the cell capacity), which ensures a rigorous assessment of the material’s stability under practical charging conditions. As shown in Fig. 7D, the cycling stability of the 3.5%SOC1022@96.5%Graphite sample exhibited stable charge–discharge behavior, and the capacity retention values after 256 cycles are 80%. Subsequently, electrochemical impedance spectroscopy (EIS) was used to understand the charge transfer behavior of sample SOC1022 before cycling, as shown in Fig. S15. It can be seen that the Nyquist plot shows a depressed semicircle in the high-frequency region and an inclined straight line in the low-frequency region.

**Fig. 8.**
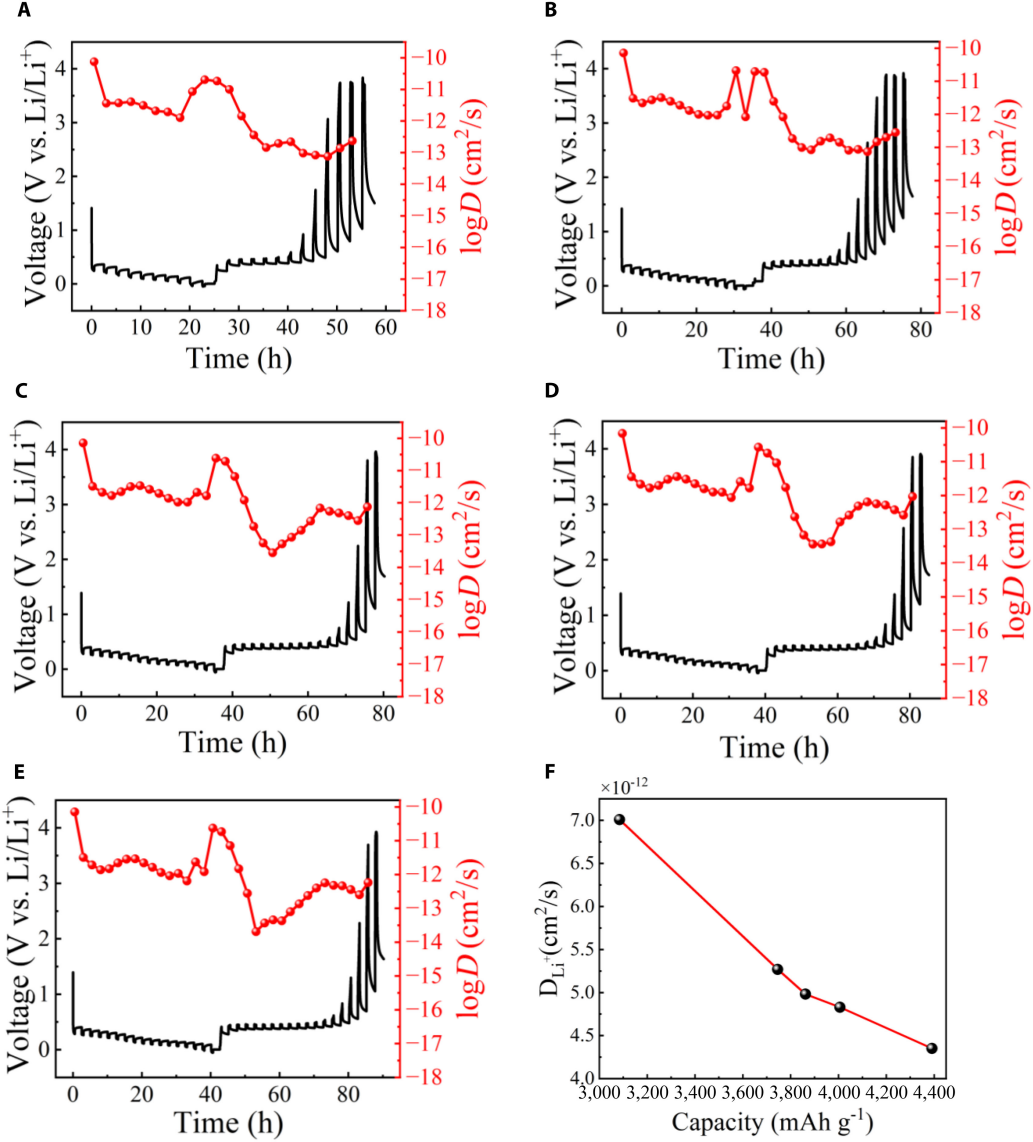
Li^+^ diffusion coefficients of the silicon–carbon anode. (A) GITT curves and corresponding Li^+^ diffusivity of the sample with an initial discharge specific capacity of 3,083.84 mAh g^−1^. (B) GITT curves and corresponding Li^+^ diffusivity of sample with an initial discharge specific capacity of 3,745.60 mAh g^−1^. (C) GITT curves and corresponding Li^+^ diffusivity of sample with an initial discharge specific capacity of 3,861.82 mAh g^−1^. (D) GITT curves and corresponding Li^+^ diffusivity of sample with an initial discharge specific capacity of 4,005.54 mAh g^−1^. (E) GITT curves and corresponding Li^+^ diffusivity of sample with an initial discharge specific capacity of 4,390.43 mAh g^−1^. (F) The variation of Li^+^ average diffusion coefficient with an initial discharge specific capacity in the GITT curves in (A) to (E).

The galvanostatic intermittent titration technique (GITT) was introduced to investigate the detailed lithium-ion transport [37,38]. The corresponding lithium-ion diffusion coefficient (D_Li+_) is calculated by Eq. (1) [39].

$$D_{{\mathrm{Li}}^{+}}=\frac{4}{\pi \tau }{\left(\frac{m_{B}V_{m}}{M_{B}S}\right)}^{2}{\left(\frac{∆E_{s}}{∆E_{\tau }}\right)}^{2}\;$$ (1)

where *τ* is the current pulse duration; *m*_B_, *M*_B_, and *V*_M_ are the mass, molar mass, and molar volume of silicon–carbon, respectively; *S* is the interfacial area of electrode–electrolyte; Δ*E*_s_ is the difference in the open circuit voltage measured at the end of 2 sequential open-circuit relaxation steps; and Δ*E_τ_* is the total voltage difference during the constant current pulse. Figure 8 shows the GITT curves of silicon–carbon anode at room temperature (tested at 1 A g^−1^, pulse time 30 min, and relaxation time 120 min). In Fig. 8A, the GITT curve of the silicon–carbon anode with an initial discharge specific capacity of 3,083.84 mAh g^−1^ is shown, and the average lithium-ion diffusion coefficient is 7.01 × 10^−12^ cm^2^ s^−1^. In Fig. 8B, the GITT curve of the silicon–carbon anode with an initial discharge specific capacity of 3,745.60 mAh g^−1^ is shown, and the average lithium-ion diffusion coefficient is 5.27 × 10^−12^ cm^2^ s^−1^. In Fig. 8C, the GITT curve of the silicon–carbon anode with an initial discharge specific capacity of 3,861.82 mAh g^−1^ is shown, and the average lithium-ion diffusion coefficient is 4.98 × 10^−12^ cm^2^ s^−1^. In Fig. 8D, the GITT curve of the silicon–carbon anode with an initial discharge specific capacity of 4,005.54 mAh g^−1^ is shown, and the average lithium-ion diffusion coefficient is 4.83 × 10^−12^ cm^2^ s^−1^. In Fig. 8E, the GITT curve of the silicon–carbon anode with an initial discharge specific capacity of 4,390.43 mAh g^−1^ is shown, and the average lithium-ion diffusion coefficient is 4.35 × 10^−12^ cm^2^ s^−1^. Figure 8F shows the variation of lithium-ion average diffusion coefficient with first discharge specific capacity in Fig. 8A to E GITT curves. The average diffusion coefficient decreases as the first discharge specific capacity increases. This counterintuitive phenomenon can be attributed to the defect-rich structure. As the defect density and specific capacity increase, while the defect structure enables ultrahigh storage capacity, the suppressed individual ion mobility imposes a kinetic limitation under high-current (high-rate) conditions, leading to the observed capacity decay. We also notice that one possible and conventional explanation is that the electrode surface gradually becomes partially passivated by irreversible solid-electrolyte interphase layers, which could hinder Li^+^ transport and reduce the measured diffusivity. Further experimental and theoretical investigations are required to rigorously verify and quantify the proposed transport mechanism.

To quantitatively predict the initial CE and initial discharge specific capacity of silicon–carbon composite anodes, a multilayer perceptron (MLP) model was developed [40]. The model was trained and validated using a dataset consisting of 96 experimentally measured synthesis–performance pairs, where each data point corresponds to a specific electrode formulation and its electrochemical performance. The input features comprised 5 primary compositional variables: (a) the mass fraction of active silicon–carbon composite, (b) carbon black, (c) conductive carbon additive, and (d) PVDF binder, as well as (e) the type of the conductive carbon additive. We introduced a second-order feature crossing strategy [41,42] before training to better capture complex interactions among material parameters. Specifically, we calculated all pairwise element-wise (Hadamard) products [43] of the original input features. This operation enriches the input space with nonlinear interaction terms without introducing additional model parameters. Mathematically, given an original input vector $x=\left[x_{1},x_{2},\ldots ,x_{n}\right]$, the Hadamard product generates new features of the form $x_{i}\cdot x_{j}$ for all *i* < *j*, resulting in a total of $n\left(n-1\right)/2$ additional features. In our case, the 5 original features were expanded to include 10 additional second-order features, yielding a total of 15 inputs to the neural network. This augmentation enables the model to learn nonlinear combinatorial effects among the formulation components, which are often critical in governing electrochemical performance, yet are difficult to capture using only first-order descriptors. The training results without feature crosses are shown in Fig. S16.

**Fig. 9.**
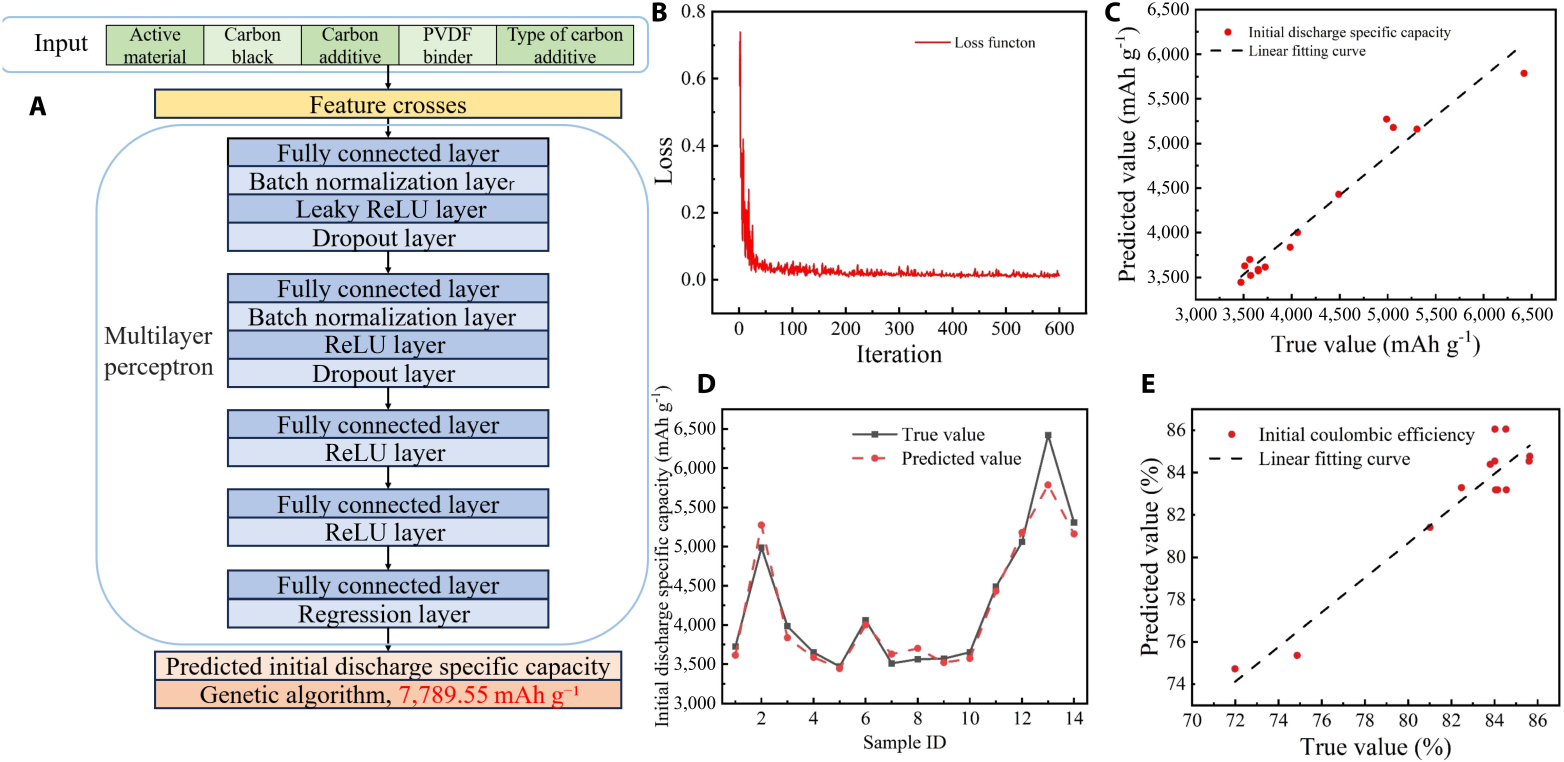
Results of AI prediction. (A) Diagram of the network structure. (B) Loss function curve. (C) Comparison between predicted and true values of the initial discharge specific capacity. (D) Error analysis for the prediction of the initial discharge specific capacity. (E) Comparison between predicted and true values of the initial CE.

The dataset was then randomly partitioned into training (70%), validation (15%), and test (15%) subsets. All features and targets were standardized using *z*-score normalization to facilitate stable and efficient model training. The neural network architecture comprised a feature input layer followed by 4 fully connected hidden layers with 512, 256, 128, and 64 neurons, respectively, as shown in Fig. 9A. The first 2 layers were regularized using dropout (rates of 30% and 20%) and batch normalization, with leaky ReLU and ReLU activation functions applied sequentially to introduce nonlinearity and avoid vanishing gradient effects. A final regression layer was used to output a continuous prediction of the target property. In addition, a custom warm-up learning rate schedule was implemented to stabilize training using stochastic gradient descent with momentum (SGDM) [44] with a gradually increasing learning rate during the initial epochs. The model was trained for 300 epochs with a mini-batch size of 32 and an initial learning rate of 1 × 10^−2^. The loss function curve for 600 iterations is shown in Fig. 9B.

After training, model performance was quantitatively evaluated on the independent test set using multiple standard regression metrics, including the root-mean-square error (RMSE), mean absolute error (MAE), and coefficient of determination (*R*^2^). For the prediction of initial discharge specific capacity, the model achieved an RMSE of 207.98 mAh g^−1^, an MAE of 145.14 mAh g^−1^, and an *R*^2^ of 0.94 for the initial discharge capacity. For the initial CE, the RMSE and MAE were 1.25% and 1.08%, respectively, with an *R*^2^ of 0.90. As shown in Fig. 9C, the predicted values of initial discharge capacity correlate strongly with experimental results, with 85.71% of predictions falling within a 5% error margin, enhanced by supplementing the charge and discharge data of the second cycle as input. Figure 9D presents a sample-wise comparison for discharge capacity. Meanwhile, Fig. 9E compares predicted and experimental values for CE, where the model achieves 100% prediction accuracy within a 5% error threshold, highlighting its robustness and consistency. Subsequently, the trained MLP model was integrated with a genetic algorithm (GA) [45] framework to obtain the theoretical maximum initial discharge specific capacity and initial CE. While the MLP provides accurate and efficient predictions of electrochemical properties from given input features, it alone does not offer a mechanism for systematically identifying the optimal combinations of input parameters. GA, a global optimization method inspired by natural evolution, is particularly well-suited for navigating high-dimensional, nonlinear, and nonconvex spaces that often characterize materials design problems. By using the MLP as a fast surrogate evaluator within the GA framework, the search process can efficiently converge toward the maximum target properties. This synergistic integration of MLP with GA enables the discovery of theoretical performance limits. The GA was configured with a population size of 100 and a maximum of 200 generations, subject to both linear equality and nonlinear inequality constraints to ensure physically feasible input formulations. The predicted maximum initial discharge specific capacity of 7,789.55 mAh g^−1^ was achieved, where the mass fractions of the 4 key continuous input parameters, namely, the mass fraction of active silicon–carbon composite, carbon black, conductive carbon additive, and PVDF binder, were 81.7%, 10.4%, 2.4%, and 5.3%, respectively. In contrast, the maximum predicted initial CE of 88.45% corresponded to a different optimized formulation, with the 4 input parameters having values of 73.7%, 8.3%, 17.6%, and 0.3%, respectively. These results demonstrate that the formulations yielding the highest specific capacity differ from those achieving the highest initial CE, underscoring an intrinsic trade-off between these 2 performance metrics and highlighting the potential of data-driven inverse design strategies in accelerating battery materials development.

## Conclusion

In summary, we report ultrahigh-capacity silicon–carbon composite anodes exhibiting lithium-storage capacities exceeding 6,500 mAh g^−1^, substantially surpassing the theoretical limit of silicon. We have achieved the capacity over 5,000 mAh g^−1^ consistently, which reveals the reproducibility of the experiments. Moreover, the initial CE can be effectively optimized by selecting appropriate lithium battery electrolytes. For example, sample SOC1205-3 using 1.0 M LiDFOB in EC:DMC = 1:1 vol% electrolyte delivers an initial discharge capacity of 5,294.88 mAh g^−1^ with a high CE of 90.96%. Furthermore, an MLP combined with GA predicts a theoretical maximum initial discharge specific capacity of 7,789.55 mAh g^−1^. The relatively low initial CE observed in the highest-capacity samples remains a crucial challenge for real-world applications. Our ongoing research focuses on optimizing surface chemistry, tailoring defect density, and engineering Si–C interfaces for improving the CE. Nevertheless, the defect-engineered high-capacity anodes may still find utility in specialized or high-energy-demand scenarios, such as aerospace systems, drones, emergency power units, or scientific instruments, where short-cycle or single-use operation is acceptable.

## Materials and Methods

### Materials fabrication

The silicon–carbon composites are synthesized via a controlled CVD process. In a typical procedure, a silicon-containing precursor (commercially available silane) was co-fed with an oxygen-containing gas into a low-pressure (<10 Pa) reaction chamber under a protective atmosphere. The silicon growth was conducted on a carbon matrix composed of 3–10 layers of graphene, enabling the formation of defect-rich silicon directly on the graphene surface. The system temperature was gradually increased to a designated reaction temperature, and maintained for a predetermined duration to facilitate the initial formation of a silicon-oxygen matrix. Subsequently, a gaseous carbon source (CH_4_), hydrogen, and a trace amount of oxygen of 10 sccm were introduced into the system. Three samples were synthesized with varying carbon source contents during the reaction process. The sample designated SOC1022 was prepared with ~10% carbon content, SOC1205 with ~20%, and SOC1218 with ~35%. The reactor was then cooled to room temperature at 50 °C/min. The concurrent presence of trace oxygen during the carbonization stage was found to enhance the structural and functional properties of the material, promoting the formation of a homogeneously bonded network with improved performance characteristics.

### Characterization methods

SEM measurements were collected using a Hitachi S-4800 (10 kV). HAADF images were acquired using aberration corrected STEM (HF5000, Hitachi) operated at the acceleration voltage of 200 kV. XPS measurements were performed using an ESCALAB 250Xi spectrometer. Raman spectra were recorded using an inVia Raman microscope.

### Electrochemical characterization

The working electrodes were made by a typical slurry method with active materials (2-dimensional silicon–carbon), conductive additive (Ketjen black), and a PVDF binder with a mass ratio of 8:1:1 and dispersed in N-methyl-2-pyrrolidone (NMP). The samples are dispersed in NMP by sonication, through which the uniformity is improved. After casting onto a 15-μm-thick Cu foil and drying at 80 °C in a vacuum oven for 12 h, the samples were cut into 113.04 mm^2^ circular disks with a mass loading of ~1–2 mg cm^−2^. In an Ar-filled glovebox, these working electrodes were assembled into type 2032 coin cells with the glass microfiber filter separator (Whatman, GF/D) and Li metal as the counter/reference electrode (half-cell). Subsequently, 100 μl of 1.0 M LiPF_6_ in EC:DEC = 1:1 vol% was added as the electrolyte with full wetting of both working and counter electrode surfaces. The galvanostatic charge/discharge test was performed between 0.005 and 1.5 V. The GITT method was used to measure D_Li+_ (testing at 1 A g^−1^, pulse time 30 min, and relaxation time 120 min) by the galvanostatic charge/discharge test. EIS measurements were performed using a CHI760e electrochemical workstation with an amplitude of 5 mV in the frequency range of 10^5^ to 10^−2^ Hz. CV measurements were performed at 0.5 mV s^−1^ scan rates in a voltage range of 0.005 to 1.5 V.

## Data Availability

All data needed to evaluate the conclusions in the paper are present in the paper or the Supplementary Materials.
