# Supplementary material for: Super High Capacity of Lithium Battery Silicon–Carbon Anode over 6,500 mAh g−1
Source: Research (Wash D C). 2026 Mar 13;9:1179. doi: 10.34133/research.1179 (PMC12982894; doi:10.34133/research.1179)
Supplement: Supplementary 1 — Tables S1 to S3 Figs. S1 to S16 [file research.1179.f1.zip › SI-Super lithium anode.docx]

**Supplementary Information**

**Super high capacity of lithium battery silicon-carbon anode over 6500 mAh g^-1^**

***Shisheng Lin***^1,2,3*^***, Minhui Yang***^1^***, Zhuang Zhao***^4,3^***, Mingjia Zhi***^4^***, Xiaokai Bai***^1^***,*** ***Kangchen Xiong***^1^

**^1^**College of Information Science and Electronic Engineering, Zhejiang University, Hangzhou, 310027, P. R. China.

^2^State Key Laboratory of Extreme Photonics and Instrumentation, Zhejiang University, Hangzhou, 310027, P. R. China.

^3^Zhejiang DiManXi Technology Co., Ltd., Zhejiang, China

^4^School of Materials Science and Engineering, Zhejiang University, Hangzhou, 310027, P. R. China.

*Address correspondence to: [shishenglin@zju.edu.cn](mailto:shishenglin@zju.edu.cn).

Table S1. The initial discharge specific capacity and Coulomb efficiency of 36 samples in Fig. S2-S7.

Table S2. The mass of the samples in the main text.

Table S3. The mass of the samples in the supporting information.

Fig. S1. Electrochemical characterization of electrolyte-optimized samples.

Fig. S2. Electrochemical characterization of high specific capacity silicon-carbon composite anodes upon cycling for sample SOC1022-4 to SOC1022-7 and SOC1205-4 to SOC1205-5.

Fig. S3. Electrochemical characterization of high specific capacity silicon-carbon composite anodes upon cycling for sample SOC1205-6 to SOC1205-11.

Fig. S4. Electrochemical characterization of high specific capacity silicon-carbon composite anodes upon cycling for sample SOC1205-12 to SOC1205-17.

Fig. S5. Electrochemical characterization of high specific capacity silicon-carbon composite anodes upon cycling for sample SOC1218-7 to SOC1218-12.

Fig. S6. Electrochemical characterization of high specific capacity silicon-carbon composite anodes upon cycling for sample SOC1218-13 to SOC1218-18.

Fig. S7. Electrochemical characterization of high specific capacity silicon-carbon composite anodes upon cycling for sample SOC1218-19 to SOC1218-24.

Fig. S8. Electrochemical characterization of silicon-carbon composite anodes with a nominal capacity of 1905.5 mAh g^-1^.

Fig. S9. dQ/dV curves of SOC 1205 electrode.

Fig. S10. dQ/dV curves of SOC 1218 electrode.

Fig. S11. PL spectroscopy of silicon-carbon anodes.

Fig. S12. SEM images of the electrode.

Fig. S13. Galvanostatic charge-discharge profiles of the blank cell.

Fig. S14. XPS analysis of silicon-carbon composite before and after cycling.

Fig. S15. The electrochemical impedance spectroscopy measurement.

Fig. S16. Training results without feature crosses.

Supplemental File 1. The original current-time-voltage data of sample SOC1218.

1. **Electrochemical performance**

**Electrochemical performance of the silicon-carbon composite anode for lithium-ion batteries.** The electrochemical performance was thoroughly analyzed and tested by assembling the active material electrode with metallic lithium into button-type half-cells, in order to assess their potentials as lithium-ion batteries anode. As shown in Extended Data Table 1, the initial discharge specific capacity ranges from 3000 to 7000 mAh g^-1^, and can reach up to 6621.43 mAh g^-1^ (the initial CE is 68.38%, SOC1022-4) and the initial CE ranges from 66 to 86%, and can reach up to 86.05% (the initial discharge specific capacity is 3777.10 mAh g^-1^, SOC1218-22).

Extended Data Table 1. The initial discharge specific capacity and Coulombic efficiency of 36 samples in Figures S2-S7.

| Sample name | The initial discharge specific capacity (mAh g^-1^) | Coulombic efficiency(%) | Sample name | The initial discharge specific capacity (mAh g^-1^) | Coulombic efficiency(%) |
| --- | --- | --- | --- | --- | --- |
| SOC1022-4 | 6621.43 | 68.38 | SOC1218-7 | 4988.61 | 84.55 |
| SOC1022-5 | 6430.83 | 70.89 | SOC1218-8 | 4925.38 | 85.44 |
| SOC1022-6 | 6420.55 | 71.98 | SOC1218-9 | 4488.83 | 84.52 |
| SOC1022-7 | 6263.43 | 66.22 | SOC1218-10 | 4427.47 | 83.19 |
| SOC1205-4 | 5906.84 | 76.66 | SOC1218-11 | 4329.98 | 83.15 |
| SOC1205-5 | 5852.06 | 69.90 | SOC1218-12 | 4285.08 | 82.16 |
| SOC1205-6 | 5767.82 | 76.21 | SOC1218-13 | 4190.59 | 82.42 |
| SOC1205-7 | 5749.13 | 76.84 | SOC1218-14 | 4101.79 | 83.79 |
| SOC1205-8 | 5712.85 | 79.16 | SOC1218-15 | 4083.67 | 81.29 |
| SOC1205-9 | 5469.63 | 73.48 | SOC1218-16 | 4061.35 | 84.01 |
| SOC1205-10 | 5382.63 | 76.97 | SOC1218-17 | 4047.97 | 84.92 |
| SOC1205-11 | 5377.34 | 76.09 | SOC1218-18 | 4025.33 | 83.88 |
| SOC1205-12 | 5304.81 | 84.02 | SOC1218-19 | 4000.01 | 83.59 |
| SOC1205-13 | 5293.50 | 72.34 | SOC1218-20 | 3907.89 | 85.01 |
| SOC1205-14 | 5274.00 | 76.48 | SOC1218-21 | 3894.77 | 85.60 |
| SOC1205-15 | 5189.14 | 77.59 | SOC1218-22 | 3777.10 | 86.05 |
| SOC1205-16 | 5151.20 | 74.57 | SOC1218-23 | 3674.36 | 85.82 |
| SOC1205-17 | 5046.74 | 72.61 | SOC1218-24 | 3653.98 | 85.27 |

Table S2. The mass of the samples in the main text.

| Sample name | The absolute mass (mg) |
| --- | --- |
| SOC1022-1 | 13.865 |
| SOC1022-2 | 13.743 |
| SOC1022-3 | 13.872 |
| SOC1205-1 | 13.321 |
| SOC1205-2 | 13.678 |
| SOC1205-3 | 13.845 |
| SOC1218-1 | 13.912 |
| SOC1218-2 | 14.036 |
| SOC1218-3 | 13.869 |
| SOC1218-4 | 13.989 |
| SOC1218-5 | 13.748 |
| SOC1218-6 | 13.852 |

Table S3. The mass of the samples in the supporting information.

| Sample name | The absolute mass (mg) | Sample name | The absolute mass (mg) |
| --- | --- | --- | --- |
| SOC1022-4 | 13.867 | SOC1218-7 | 13.759 |
| SOC1022-5 | 13.687 | SOC1218-8 | 14.043 |
| SOC1022-6 | 13.748 | SOC1218-9 | 13.699 |
| SOC1022-7 | 13.693 | SOC1218-10 | 13.947 |
| SOC1205-4 | 13.469 | SOC1218-11 | 13.896 |
| SOC1205-5 | 13.367 | SOC1218-12 | 14.181 |
| SOC1205-6 | 13.481 | SOC1218-13 | 13.967 |
| SOC1205-7 | 13.743 | SOC1218-14 | 13.941 |
| SOC1205-8 | 13.627 | SOC1218-15 | 13.755 |
| SOC1205-9 | 13.488 | SOC1218-16 | 14.278 |
| SOC1205-10 | 13.575 | SOC1218-17 | 14.005 |
| SOC1205-11 | 13.461 | SOC1218-18 | 13.951 |
| SOC1205-12 | 13.379 | SOC1218-19 | 13.714 |
| SOC1205-13 | 13.657 | SOC1218-20 | 13.955 |
| SOC1205-14 | 13.413 | SOC1218-21 | 13.859 |
| SOC1205-15 | 13.569 | SOC1218-22 | 13.928 |
| SOC1205-16 | 13.704 | SOC1218-23 | 14.023 |
| SOC1205-17 | 13.587 | SOC1218-24 | 13.881 |

The absolute mass of each electrode is provided in Table S2 and Table S3. Each sample was weighed three times independently to ensure measurement accuracy and reproducibility. All measurements were conducted using an Electronic Balance XPR36C/AC.

**
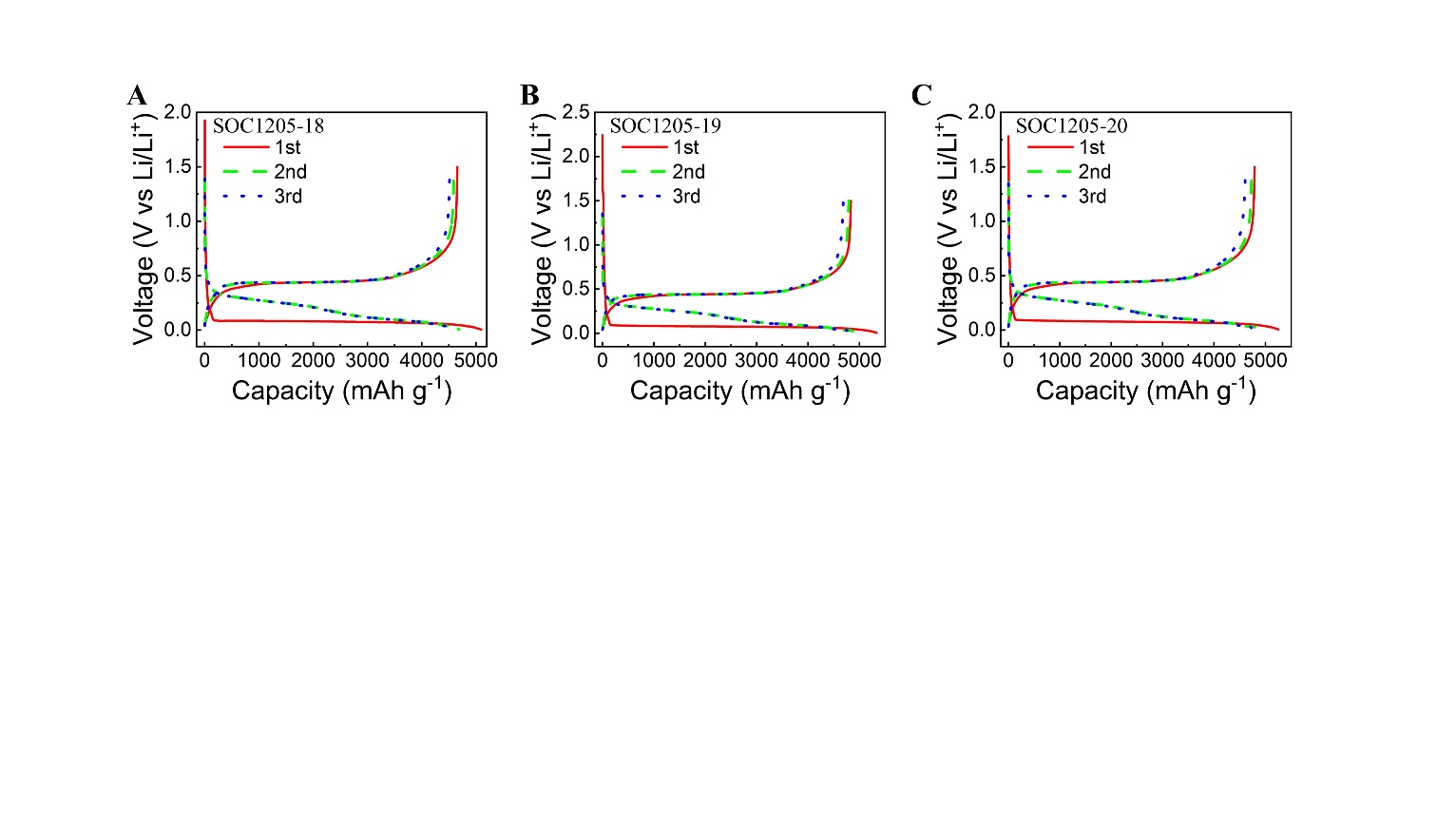
**

**S. 1. Electrochemical characterization of electrolyte-optimized samples.** (A) Performance of SOC 1205-18 electrode in 1.0 M LiDFOB in EC:DMC=1:1 vol% electrolyte with a CE of 91.31% and an initial discharge capacity of 5098.19 mAh g^-1^. (B) Performance of SOC 1205-19 electrode in 1.0 M LiDFOB in EC:DMC=1:1 vol% electrolyte with a CE of 90.58% and an initial discharge capacity of 5333.80 mAh g^-1^. (C) Performance of SOC 1205-20 electrode in 1.0 M LiDFOB in EC:DMC=1:1 vol% electrolyte with a CE of 91.15% and an initial discharge capacity of 5253.80 mAh g^-1^.

S.2 shows the [galvanostatic](http://www.baidu.com/link?url=2OKFsmnCjAySirII7oHJDv0oSCBlRzIjVdMe63GtdDR3cfylfvq_vUG70klZBqzE266NPt_NcHkQvDM7Focw5mBoGYdY5XUBaH7blAoUcl_) charge-discharge curves (GCD) of high specific capacity silicon-carbon samples from SOC1022-4 to SOC1022-7 and SOC1205-4 to SOC 1205-5 at a 0.1C rate for the first three cycles; the voltage window is set to 0.005-1.5V, and the current density for 1C is 4200 mA g^-1^. In S.2A, for sample SOC1022-4, the initial discharge specific capacity is 6621.43 mAh g^-1^ with a CE of 68.38%; the discharge specific capacity in the second cycle is 4318.40 mAh g^-1^ with a CE of 86.66%, and in the third cycle, the discharge specific capacity is 3587.69 mAh g^-1^ with a CE of 86.49%. In S.2B, for sample SOC1022-5, the initial discharge specific capacity is 6430.83 mAh g^-1^ with a CE of 70.89%; the discharge specific capacity in the second cycle is 4400.15 mAh g^-1^ with a CE of 90.33%, and in the third cycle, the discharge specific capacity is 3887.68 mAh g^-1^ with a CE of 92.15%. In S.2C, for sample SOC1022-6, the initial discharge specific capacity is 6420.55 mAh g^-1^ with a CE of 71.98%; the discharge specific capacity in the second cycle is 4487.62 mAh g^-1^ with a CE of 91.47%, and in the third cycle, the discharge specific capacity is 3948.16 mAh g^-1^ with a CE of 92.44%. In S.2D, for sample SOC1022-7, the initial discharge specific capacity is 6263.43 mAh g^-1^ with a CE of 66.22%; the discharge specific capacity in the second cycle is 3958.96 mAh g^-1^ with a CE of 88.31%, and in the third cycle, the discharge specific capacity is 3304.25 mAh g^-1^ with a CE of 90.33%. In S.2E, for sample SOC1205-4, the initial discharge specific capacity is 5906.84 mAh g^-1^ with a CE of 76.66%; the discharge specific capacity in the second cycle is 4496.28 mAh g^-1^ with a CE of 92.59%, and in the third cycle, the discharge specific capacity is 4042.13 mAh g^-1^ with a CE of 93.29%. And in S.2F, for sample SOC1205-5, the initial discharge specific capacity is 5852.06 mAh g^-1^ with a CE of 69.90%; the discharge specific capacity in the second cycle is 3924.72 mAh g^-1^ with a CE of 91.84%, and in the third cycle, the discharge specific capacity is 3465.30 mAh g^-1^ with a CE of 92.96%. The data in S.2 shows that the initial discharge specific capacity of our high-performance half cell is between 5800-6700 mAh g^-1^, and the highest one can reach 6621.43 mAh g^-1^ with the CE is 68.38% (SOC1022-4).

**
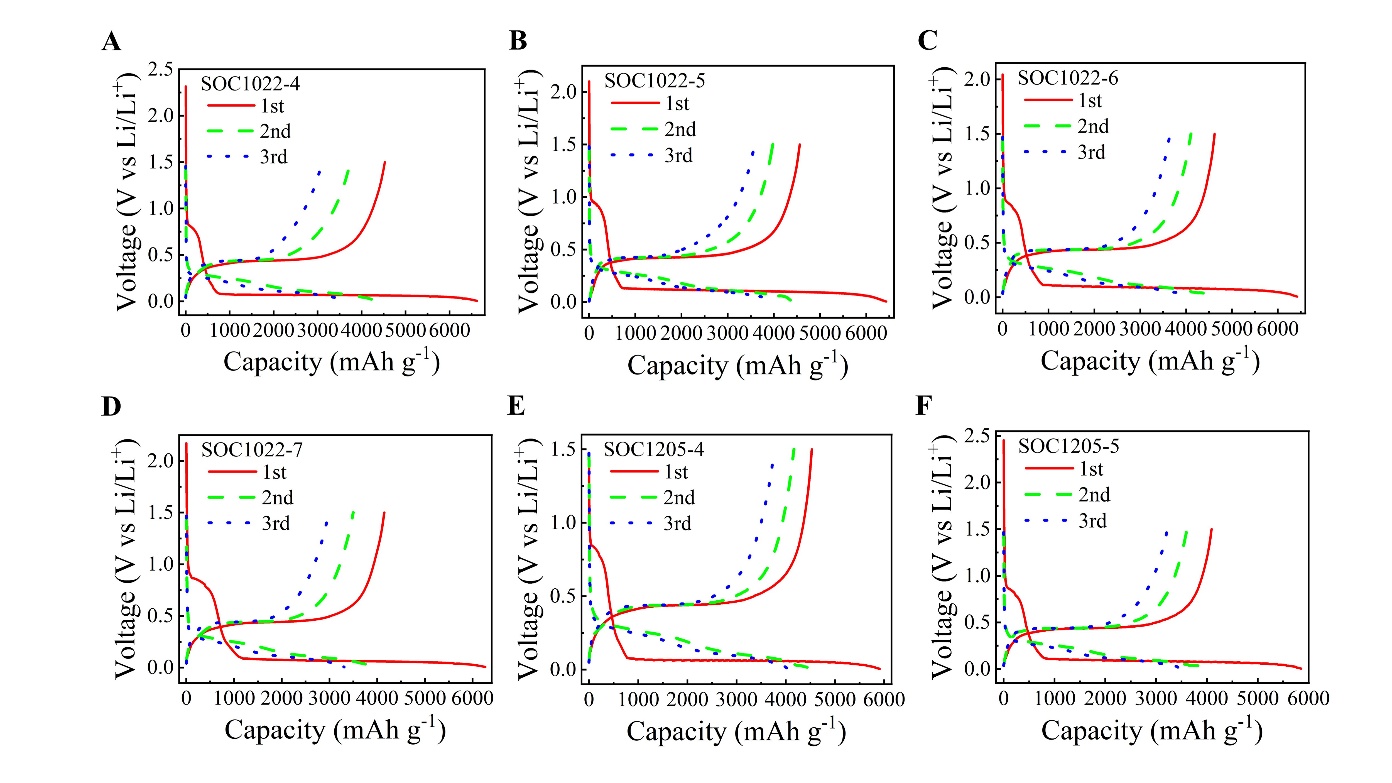
**

**S. 2.** **Electrochemical characterization of high specific capacity silicon-carbon composite anodes upon cycling.** (A) GCD at 0.42 A g^-1^ for the first three cycles for sample SOC1022-4. (B) GCD at 0.42 A g^-1^ for the first three cycles for sample SOC1022-5. (C) GCD at 0.42 A g^-1^ for the first three cycles for sample SOC1022-6. (D) GCD at 0.42 A g^-1^ for the first three cycles for sample SOC1022-7. (E) GCD at 0.42 A g^-1^ for the first three cycles for sample SOC1205-4. (F) GCD at 0.42 A g^-1^ for the first three cycles for sample SOC1205-5.

S.3 shows the GCD of high specific capacity ratio silicon-carbon samples from SOC1205-6 to SOC1205-11 at a 0.1C rate for the first three cycles; the voltage window is set to 0.005-1.5V, and the current density for 1C is 4200 mA g^-1^. In S.3A, for sample SOC1205-6, the initial discharge specific capacity is 5767.82 mAh g^-1^ with a CE of 76.21%; the discharge specific capacity in the second cycle is 4382.06 mAh g^-1^ with a CE of 92.44%, and in the third cycle, the discharge specific capacity is 3932.25 mAh g^-1^ with a CE of 93.27%. In S.3B, for sample SOC1205-7, the initial discharge specific capacity is 5749.13 mAh g^-1^ with a CE of 76.84%; the discharge specific capacity in the second cycle is 4571.98 mAh g^-1^ with a CE of 97.23%, and in the third cycle, the discharge specific capacity is 4484.95 mAh g^-1^ with a CE of 99.25%. In S.3C, for sample SOC1205-8, the initial discharge specific capacity is 5712.85 mAh g^-1^ with a CE of 79.16%; the discharge specific capacity in the second cycle is 4634.90 mAh g^-1^ with a CE of 97.19%, and in the third cycle, the discharge specific capacity is 4575.43 mAh g^-1^ with a CE of 98.69%. In S.3D, for sample SOC1205-9, the initial discharge specific capacity is 5469.63 mAh g^-1^ with a CE of 73.48%; the discharge specific capacity in the second cycle is 3918.89 mAh g^-1^ with a CE of 91.23%, and in the third cycle, the discharge specific capacity is 3464.71 mAh g^-1^ with a CE of 91.73%. In S.3E, for sample SOC1205-10, the initial discharge specific capacity is 5382.63 mAh g^-1^ with a CE of 76.97%; the discharge specific capacity in the second cycle is 4069.97 mAh g^-1^ with a CE of 94.42%, and in the third cycle, the discharge specific capacity is 3694.92 mAh g^-1^ with a CE of 95.77%. And in S.3F, for sample SOC1205-11, the initial discharge specific capacity is 5377.34 mAh g^-1^ with a CE of 76.09%; the discharge specific capacity in the second cycle is 4062.28 mAh g^-1^ with a CE of 92.34%, and in the third cycle, the discharge specific capacity is 3620.23 mAh g^-1^ with a CE of 93.10%. The data in S.3 shows that the initial discharge specific capacity of our high-performance half cell is between 5300-5800 mAh g^-1^, and the highest one can reach 5767.82 mAh g^-1^ with the CE is 76.21% (SOC1205-6).


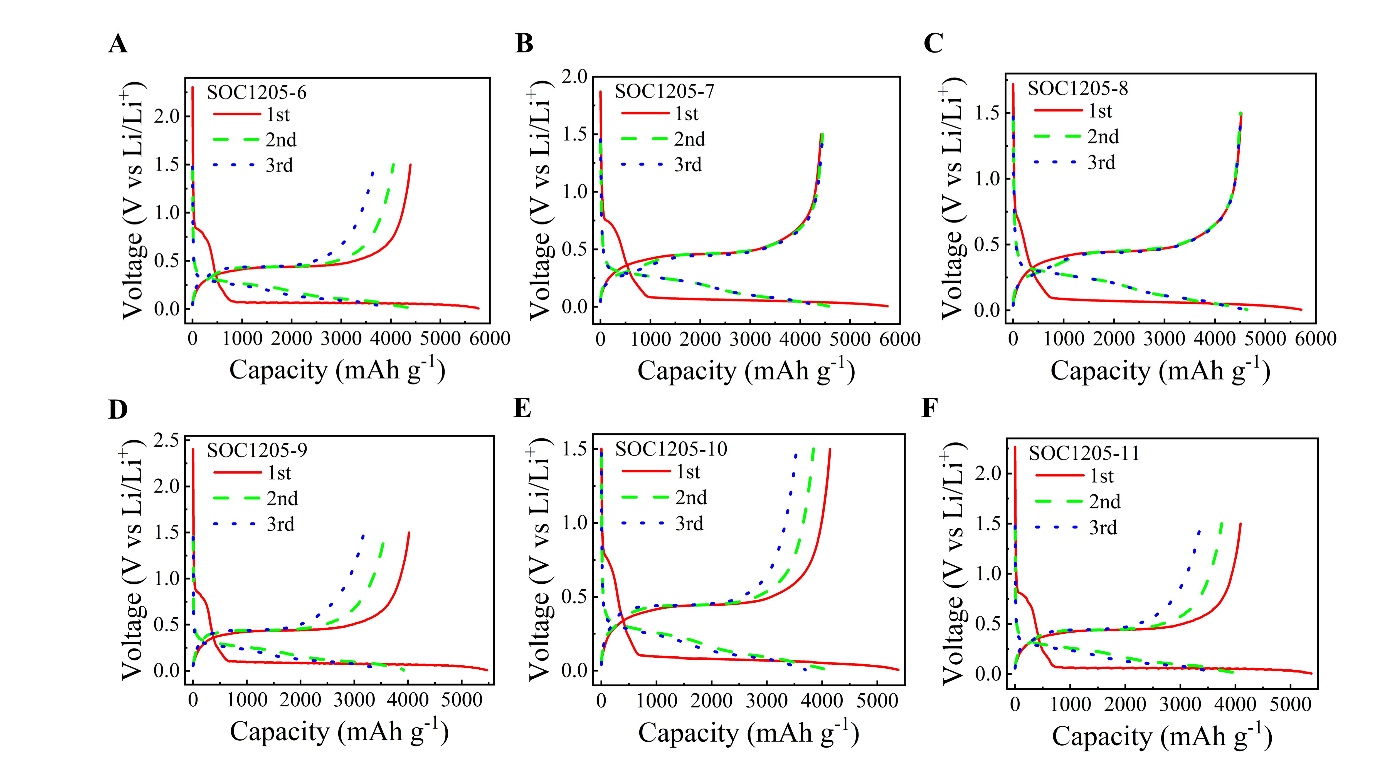


**S. 3. Electrochemical characterization of high specific capacity silicon-carbon composite anodes upon cycling.** (A) GCD at 0.42 A g^-1^ for the first three cycles for sample SOC1205-6. (B) GCD at 0.42 A g^-1^ for the first three cycles for sample SOC1205-7. (C) GCD at 0.42 A g^-1^ for the first three cycles for sample SOC1205-8. (D) GCD at 0.42 A g^-1^ for the first three cycles for sample SOC1205-9. (E) GCD at 0.42 A g^-1^ for the first three cycles for sample SOC1205-10. (F) GCD at 0.42 A g^-1^ for the first three cycles for sample SOC1205-11.

S.4 shows the GCD of high specific capacity ratio silicon-carbon samples from SOC1205-12 to SOC1205-17 at a 0.1C rate for the first three cycles; the voltage window is set to 0.005-1.5V, and the current density for 1C is 4200 mA g^-1^. In S.4A, for sample SOC1205-12, the initial discharge specific capacity is 5304.81 mAh g^-1^ with a CE of 84.02%; the discharge specific capacity in the second cycle is 4418.39 mAh g^-1^ with a CE of 95.25%, and in the third cycle, the discharge specific capacity is 4121.10 mAh g^-1^ with a CE of 96.40%. In S.4B, for sample SOC1205-13, the initial discharge specific capacity is 5293.50 mAh g^-1^ with a CE of 72.34%; the discharge specific capacity in the second cycle is 3695.05 mAh g^-1^ with a CE of 91.23%, and in the third cycle, the discharge specific capacity is 3264.82 mAh g^-1^ with a CE of 91.41%. In S.4C, for sample SOC1205-14, the initial discharge specific capacity is 5274.00 mAh g^-1^ with a CE of 76.48%; the discharge specific capacity in the second cycle is 3947.34 mAh g^-1^ with a CE of 94.37%, and in the third cycle, the discharge specific capacity is 3574.32 mAh g^-1^ with a CE of 95.69%. In S.4D, for sample SOC1205-15, the initial discharge specific capacity is 5189.14 mAh g^-1^ with a CE of 77.59%; the discharge specific capacity in the second cycle is 3980.63 mAh g^-1^ with a CE of 91.71%, and in the third cycle, the discharge specific capacity is 3547.65 mAh g^-1^ with a CE of 92.00%. In S.4E, for sample SOC1205-16, the initial discharge specific capacity is 5151.20 mAh g^-1^ with a CE of 74.57%; the discharge specific capacity in the second cycle is 3789.90 mAh g^-1^ with a CE of 93.74%, and in the third cycle, the discharge specific capacity is 3526.44 mAh g^-1^ with a CE of 94.45%. And in S.4F, for sample SOC1205-17, the initial discharge specific capacity is 5046.74 mAh g^-1^ with a CE of 72.61%; the discharge specific capacity in the second cycle is 3560.69 mAh g^-1^ with a CE of 91.43%, and in the third cycle, the discharge specific capacity is 3159.97 mAh g^-1^ with a CE of 91.93%. The data in S.4 shows that the initial discharge specific capacity of our high-performance half cell is between 5000-5400 mAh g^-1^, and the highest one can reach 5304.81 mAh g^-1^ with the CE is 84.02% (SOC1205-12).


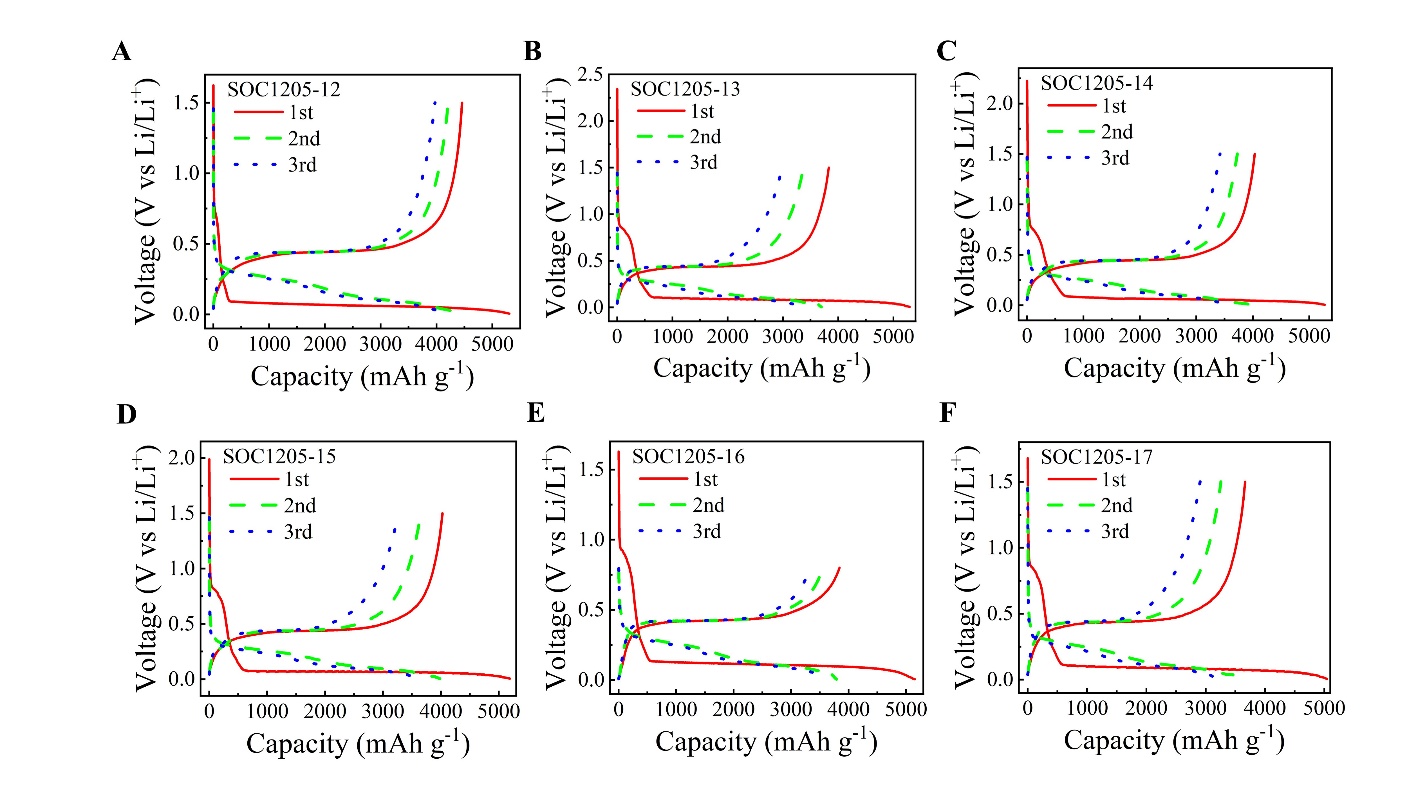


**S. 4. Electrochemical characterization of high specific capacity silicon-carbon composite anodes upon cycling.** (A) GCD at 0.42 A g^-1^ for the first three cycles for sample SOC1205-12. (B) GCD at 0.42 A g^-1^ for the first three cycles for sample SOC1205-13. (C) GCD at 0.42 A g^-1^ for the first three cycles for sample SOC1205-14. (D) GCD at 0.42 A g^-1^ for the first three cycles for sample SOC1205-15. (E) GCD at 0.42 A g^-1^ for the first three cycles for sample SOC1205-16. (F) GCD at 0.42 A g^-1^ for the first three cycles for sample SOC1205-17.

S.5 shows the GCD of high Coulombic efficiency ratio two-dimensional silicon-carbon from SOC1218-7 to SOC1218-12 at a 0.1C rate for the first three cycles; the voltage window is set to 0.005-1.5V, and the current density for 1C is 4200 mA g^-1^. In S.5A, for sample SOC1218-7, the initial discharge specific capacity is 4988.61 mAh g^-1^ with a CE of 84.55%; the discharge specific capacity in the second cycle is 4281.34 mAh g^-1^ with a CE of 97.31%, and in the third cycle, the discharge specific capacity is 4140.02 mAh g^-1^ with a CE of 97.66%. In S.5B, for sample SOC1218-8, the initial discharge specific capacity is 4925.38 mAh g^-1^ with a CE of 85.44%; the discharge specific capacity in the second cycle is 4178.82 mAh g^-1^ with a CE of 91.71%, and in the third cycle, the discharge specific capacity is 3760.46 mAh g^-1^ with a CE of 88.95%. In S.5C, for sample SOC1218-9, the initial discharge specific capacity is 4488.83 mAh g^-1^ with a CE of 84.52%; the discharge specific capacity in the second cycle is 3755.94 mAh g^-1^ with a CE of 93.99%, and in the third cycle, the discharge specific capacity is 3429.36 mAh g^-1^ with a CE of 94.38%. In S.5D, for sample SOC1218-10, the initial discharge specific capacity is 4427.47 mAh g^-1^ with a CE of 83.19%; the discharge specific capacity in the second cycle is 3657.96 mAh g^-1^ with a CE of 92.44%, and in the third cycle, the discharge specific capacity is 3333.40 mAh g^-1^ with a CE of 91.76%. In S.5E, for sample SOC1218-11, the initial discharge specific capacity is 4329.98 mAh g^-1^ with a CE of 83.15%; the discharge specific capacity in the second cycle is 3588.57 mAh g^-1^ with a CE of 92.47%, and in the third cycle, the discharge specific capacity is 3275.15 mAh g^-1^ with a CE of 91.87%. And in S.5F, for sample SOC1218-12, the initial discharge specific capacity is 4285.08 mAh g^-1^ with a CE of 82.16%; the discharge specific capacity in the second cycle is 3545.87 mAh g^-1^ with a CE of 95.58%, and in the third cycle, the discharge specific capacity is 3364.00 mAh g^-1^ with a CE of 95.68%. The data in S.5 shows the charge and discharge data with high initial CE, which ranges from 82 to 86%, and can reach up to 85.44% (the initial discharge specific capacity is 4925.38 mAh g^-1^, SOC1218-8).


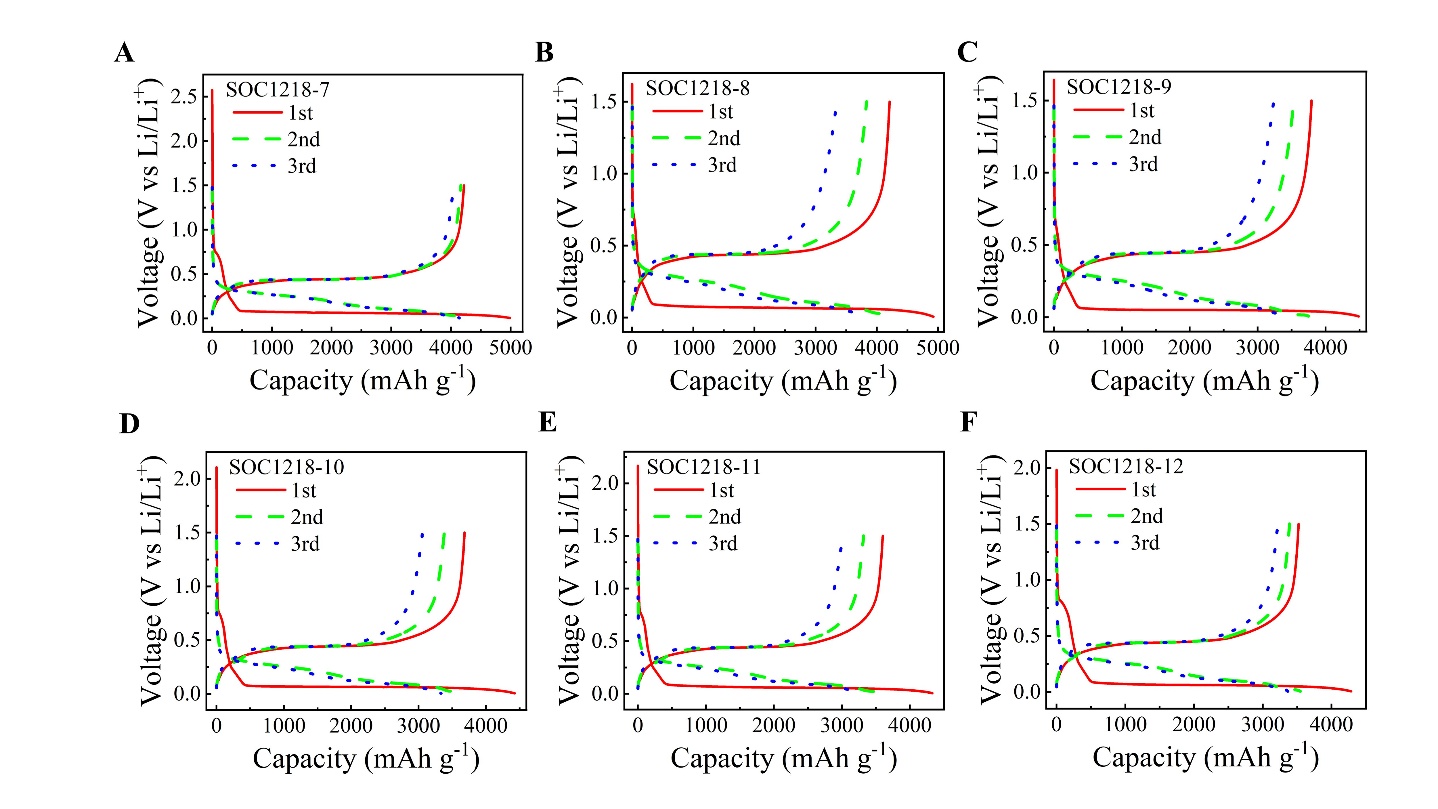


**S. 5. Electrochemical characterization of silicon-carbon composite anodes with high CE.** (A) GCD at 0.42 A g^-1^ for the first three cycles for sample SOC1218-7. (B) GCD at 0.42 A g^-1^ for the first three cycles for sample SOC1218-8. (C) GCD at 0.42 A g^-1^ for the first three cycles for sample SOC1218-9. (D) GCD at 0.42 A g^-1^ for the first three cycles for sample SOC1218-10. (E) GCD at 0.42 A g^-1^ for the first three cycles for sample SOC1218-11. (F) GCD at 0.42 A g^-1^ for the first three cycles for sample SOC1218-12.

S.6 shows the GCD of high Coulombic efficiency ratio two-dimensional silicon-carbon samples from SOC1218-13 to SOC1218-18 at a 0.1C rate for the first three cycles; the voltage window is set to 0.005-1.5V, and the current density for 1C is 4200 mA g^-1^. In S.6A, for sample SOC1218-13, the initial discharge specific capacity is 4190.59 mAh g^-1^ with a CE of 82.42%; the discharge specific capacity in the second cycle is 3472.82 mAh g^-1^ with a CE of 95.69%, and in the third cycle, the discharge specific capacity is 3283.34 mAh g^-1^ with a CE of 95.85%. In S.6B, for sample SOC1218-14, the initial discharge specific capacity is 4101.79 mAh g^-1^ with a CE of 83.79%; the discharge specific capacity in the second cycle is 3400.93 mAh g^-1^ with a CE of 91.56%, and in the third cycle, the discharge specific capacity is 3051.18 mAh g^-1^ with a CE of 88.66%. In S.6C, for sample SOC1218-15, the initial discharge specific capacity is 4083.67 mAh g^-1^ with a CE of 81.29%; the discharge specific capacity in the second cycle is 3349.17 mAh g^-1^ with a CE of 96.41%, and in the third cycle, the discharge specific capacity is 3162.01 mAh g^-1^ with a CE of 96.62%. In S.6D, for sample SOC1218-16, the initial discharge specific capacity is 4061.35 mAh g^-1^ with a CE of 84.01%; the discharge specific capacity in the second cycle is 3369.36 mAh g^-1^ with a CE of 93.70%, and in the third cycle, the discharge specific capacity is 3101.53 mAh g^-1^ with a CE of 93.57%. In S.6E, for sample SOC1218-17, the initial discharge specific capacity is 4047.97 mAh g^-1^ with a CE of 84.92%; the discharge specific capacity in the second cycle is 3355.77 mAh g^-1^ with a CE of 94.30%, and in the third cycle, the discharge specific capacity is 3074.02 mAh g^-1^ with a CE of 94.60%. And in S.6F, for sample SOC1218-18, the initial discharge specific capacity is 4025.33 mAh g^-1^ with a CE of 83.88%; the discharge specific capacity in the second cycle is 3358.62 mAh g^-1^ with a CE of 95.72%, and in the third cycle, the discharge specific capacity is 3172.85 mAh g^-1^ with a CE of 96.72%. The data in S.6 shows the charge and discharge data with high initial CE, which ranges from 81 to 85%, and can reach up to 84.92% (the initial discharge specific capacity is 4047.97 mAh g^-1^, SOC1218-17).


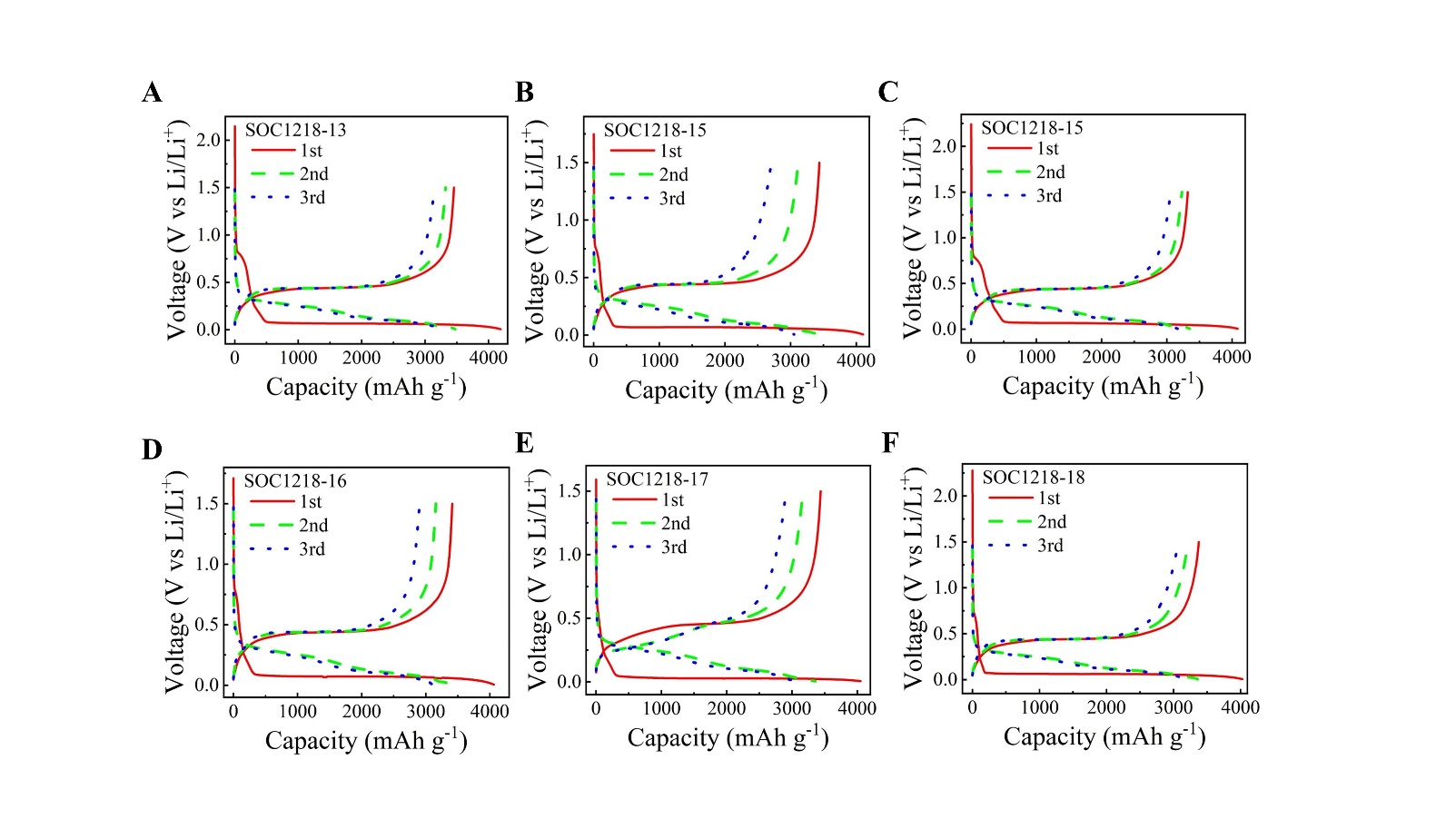


**S. 6. Electrochemical characterization of silicon-carbon composite anodes with high CE.** (A) GCD at 0.42 A g^-1^ for the first three cycles for sample SOC1218-13. (B) GCD at 0.42 A g^-1^ for the first three cycles for sample SOC1218-14. (C) GCD at 0.42 A g^-1^ for the first three cycles for sample SOC1218-15. (D) GCD at 0.42 A g^-1^ for the first three cycles for sample SOC1218-16. (E) GCD at 0.42 A g^-1^ for the first three cycles for sample SOC1218-17. (F) GCD at 0.42 A g^-1^ for the first three cycles for sample SOC1218-18.

S.7 shows the GCD of high Coulombic efficiency ratio two-dimensional silicon-carbon samples from SOC1218-19 to SOC1218-24 at a 0.1C rate for the first three cycles; the voltage window is set to 0.005-1.5V, and the current density for 1C is 4200 mA g^-1^. In S.7A, for sample SOC1218-19, the initial discharge specific capacity is 4000.01 mAh g^-1^ with a CE of 83.59%; the discharge specific capacity in the second cycle is 3375.68 mAh g^-1^ with a CE of 96.99%, and in the third cycle, the discharge specific capacity is 3294.19 mAh g^-1^ with a CE of 97.30%. In S.7B, for sample SOC1218-20, the initial discharge specific capacity is 3907.89 mAh g^-1^ with a CE of 85.01%; the discharge specific capacity in the second cycle is 3351.22 mAh g^-1^ with a CE of 95.47%, and in the third cycle, the discharge specific capacity is 3191.45 mAh g^-1^ with a CE of 96.11%. In S.7C, for sample SOC1218-21, the initial discharge specific capacity is 3894.77 mAh g^-1^ with a CE of 85.60%; the discharge specific capacity in the second cycle is 3256.18 mAh g^-1^ with a CE of 93.68%, and in the third cycle, the discharge specific capacity is 2959.82 mAh g^-1^ with a CE of 94.25%. In S.7D, for sample SOC1218-22, the initial discharge specific capacity is 3777.10 mAh g^-1^ with a CE of 86.05%; the discharge specific capacity in the second cycle is 3215.66 mAh g^-1^ with a CE of 93.97%, and in the third cycle, the discharge specific capacity is 2950.84 mAh g^-1^ with a CE of 93.66%. In S.7E, for sample SOC1218-23, the initial discharge specific capacity is 3674.36 mAh g^-1^ with a CE of 85.82%; the discharge specific capacity in the second cycle is 3136.85 mAh g^-1^ with a CE of 94.58%, and in the third cycle, the discharge specific capacity is 2938.36 mAh g^-1^ with a CE of 93.04%. And in S.7F, for sample SOC1218-24, the initial discharge specific capacity is 3653.98 mAh g^-1^ with a CE of 85.27%; the discharge specific capacity in the second cycle is 3098.35 mAh g^-1^ with a CE of 94.76%, and in the third cycle, the discharge specific capacity is 2833.64 mAh g^-1^ with a CE of 94.52%. The data in S.7 shows the charge and discharge data with high initial CE, which ranges from 83 to 87%, and can reach up to 86.05% (the initial discharge specific capacity is 3777.10 mAh g^-1^, SOC1218-22).

**
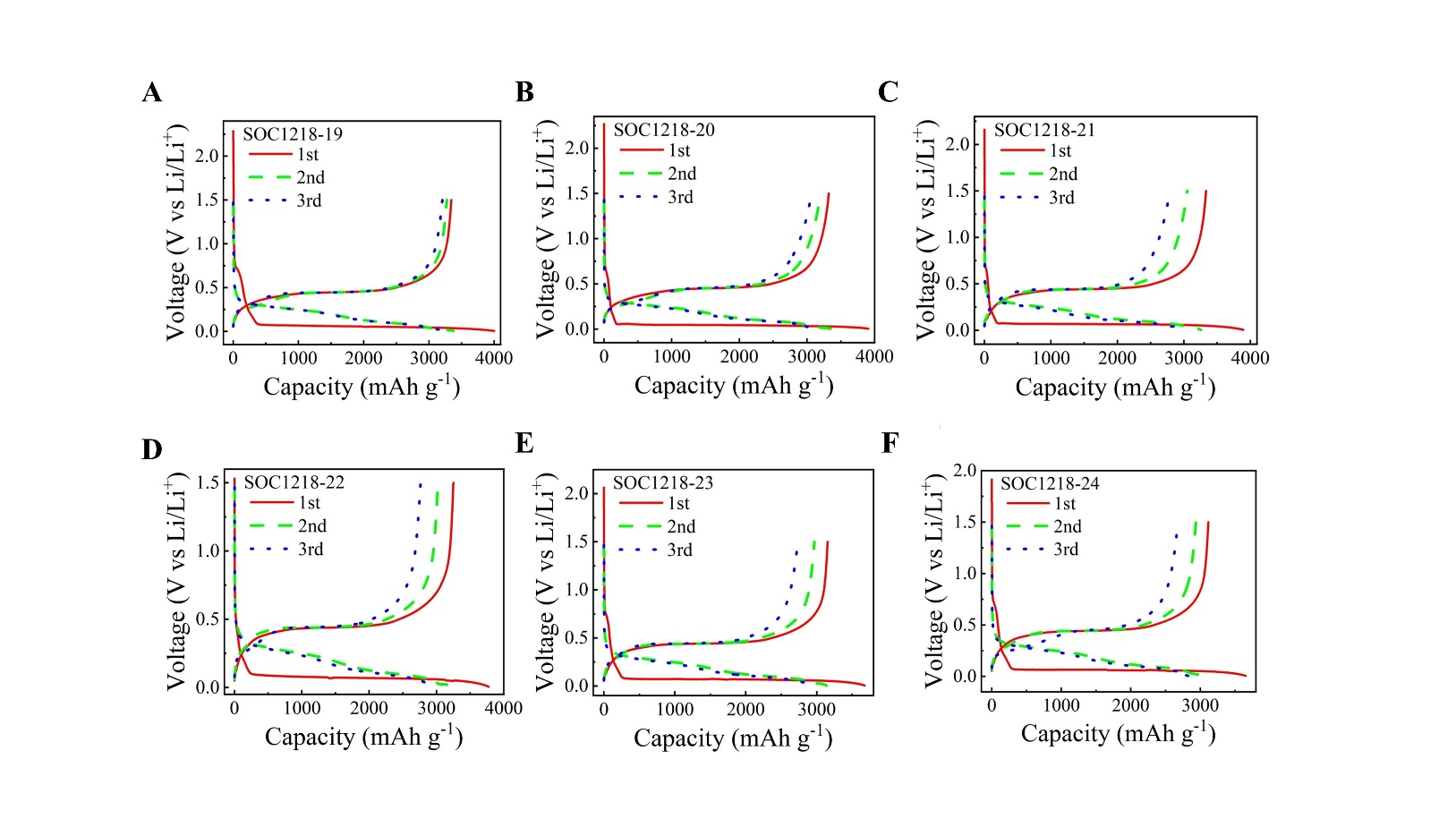
**

**S. 7. Electrochemical characterization of silicon-carbon composite anodes with high CE.** (A) GCD at 0.42 A g^-1^ for the first three cycles for sample SOC1218-19. (B) GCD at 0.42 A g^-1^ for the first three cycles for sample SOC1218-20. (C) GCD at 0.42 A g^-1^ for the first three cycles for sample SOC1218-21. (D) GCD at 0.42 A g^-1^ for the first three cycles for sample SOC1218-22. (E) GCD at 0.42 A g^-1^ for the first three cycles for sample SOC1218-23. (F) GCD at 0.42 A g^-1^ for the first three cycles for sample SOC1218-24.

**
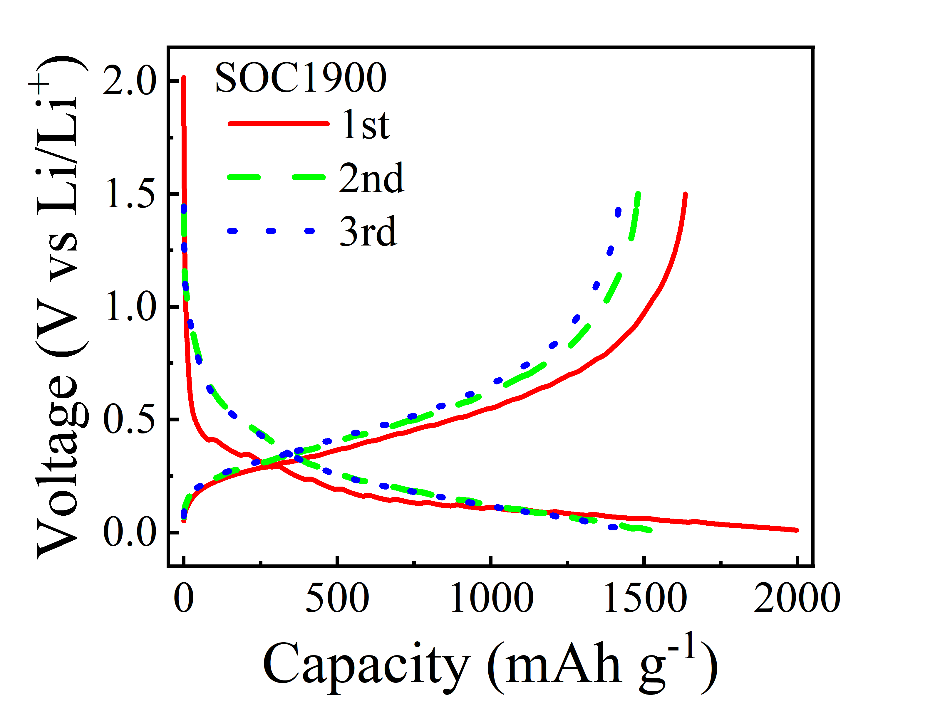
**

**S. 8.** **Electrochemical characterization of silicon-carbon composite anodes with a nominal capacity of 1905.5 mAh g^-1^.**


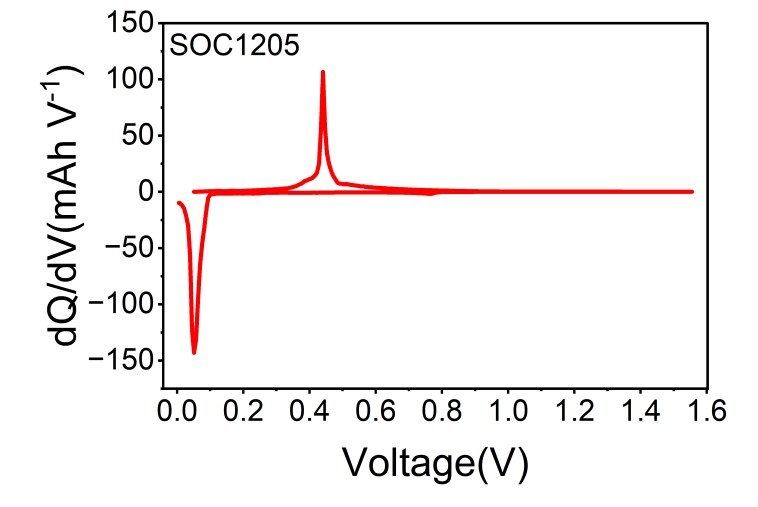


**S. 9. dQ/dV curves of SOC1205 electrode.**


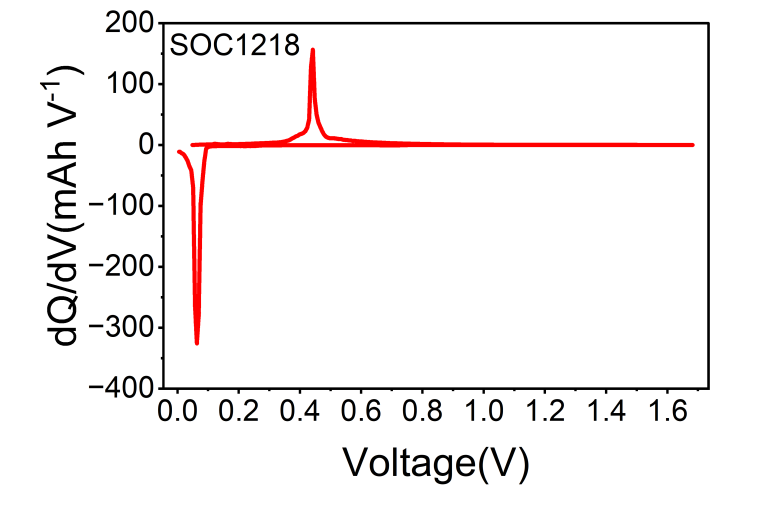


**S. 10. dQ/dV curves of SOC1218 electrode.**


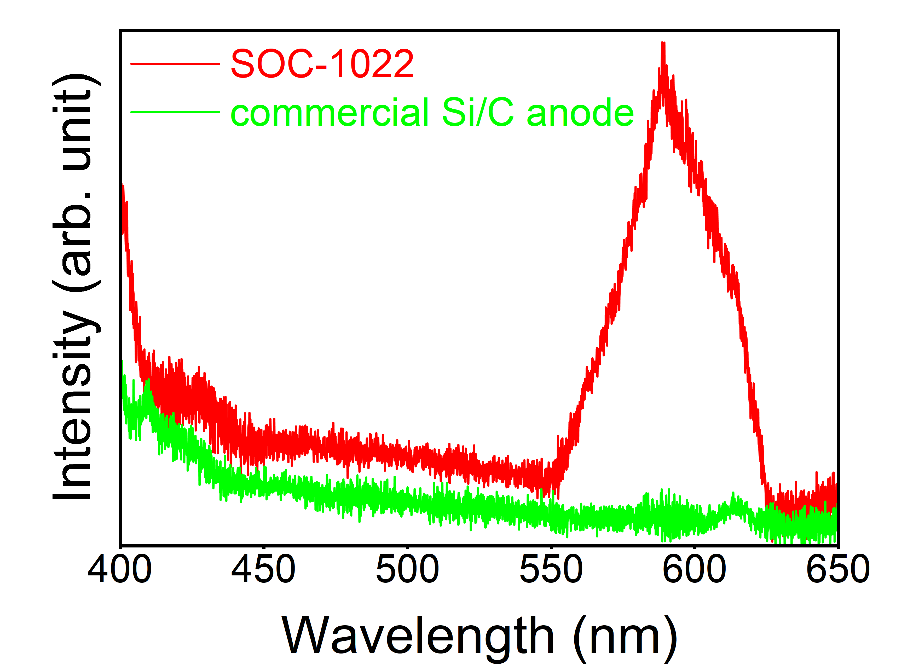


**S. 11. PL spectroscopy of silicon-carbon anodes.**


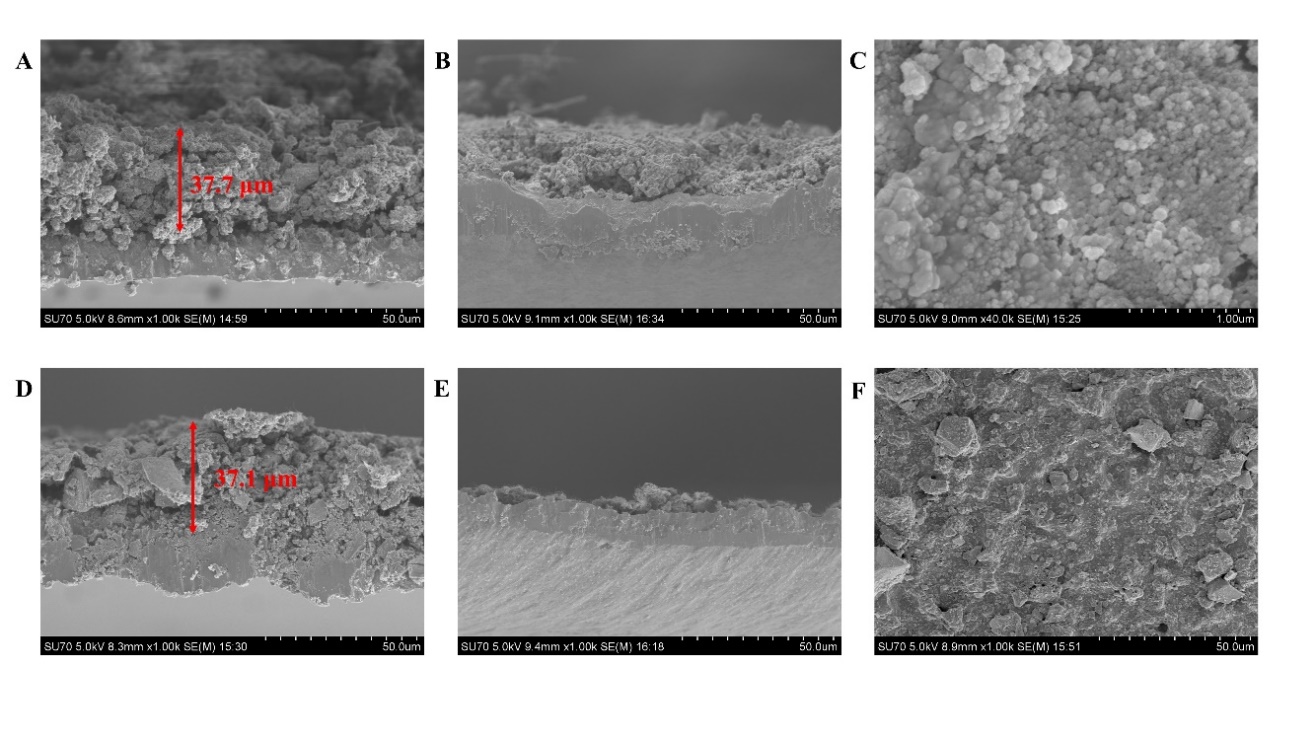


**S. 12. SEM images of the electrode. (A-C) SEM images of the electrode after cycling. (D-F) SEM images of the electrode before cycling.**

**
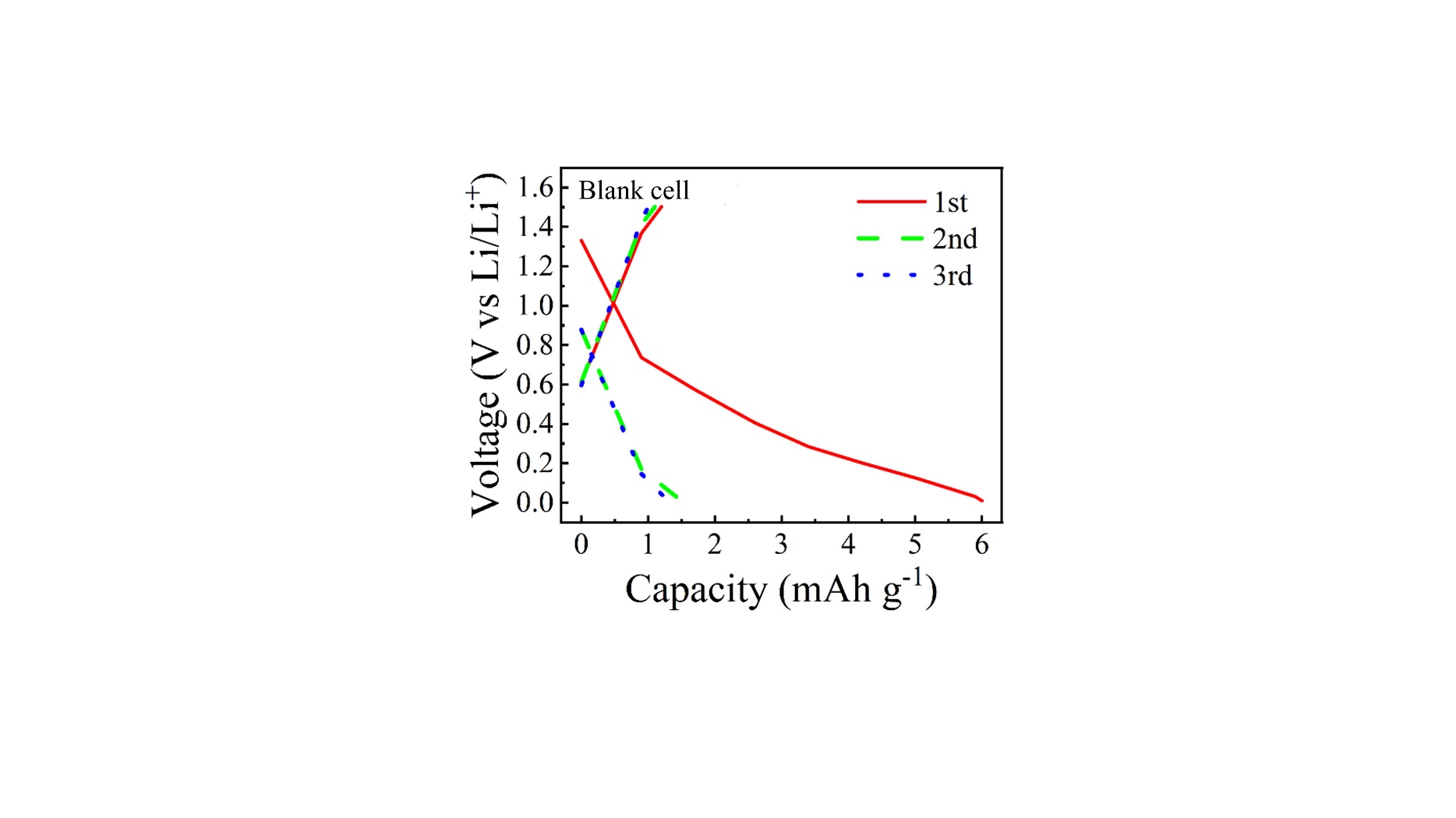
**

**S. 13.** **Galvanostatic charge-discharge profiles of the blank cell.**

**
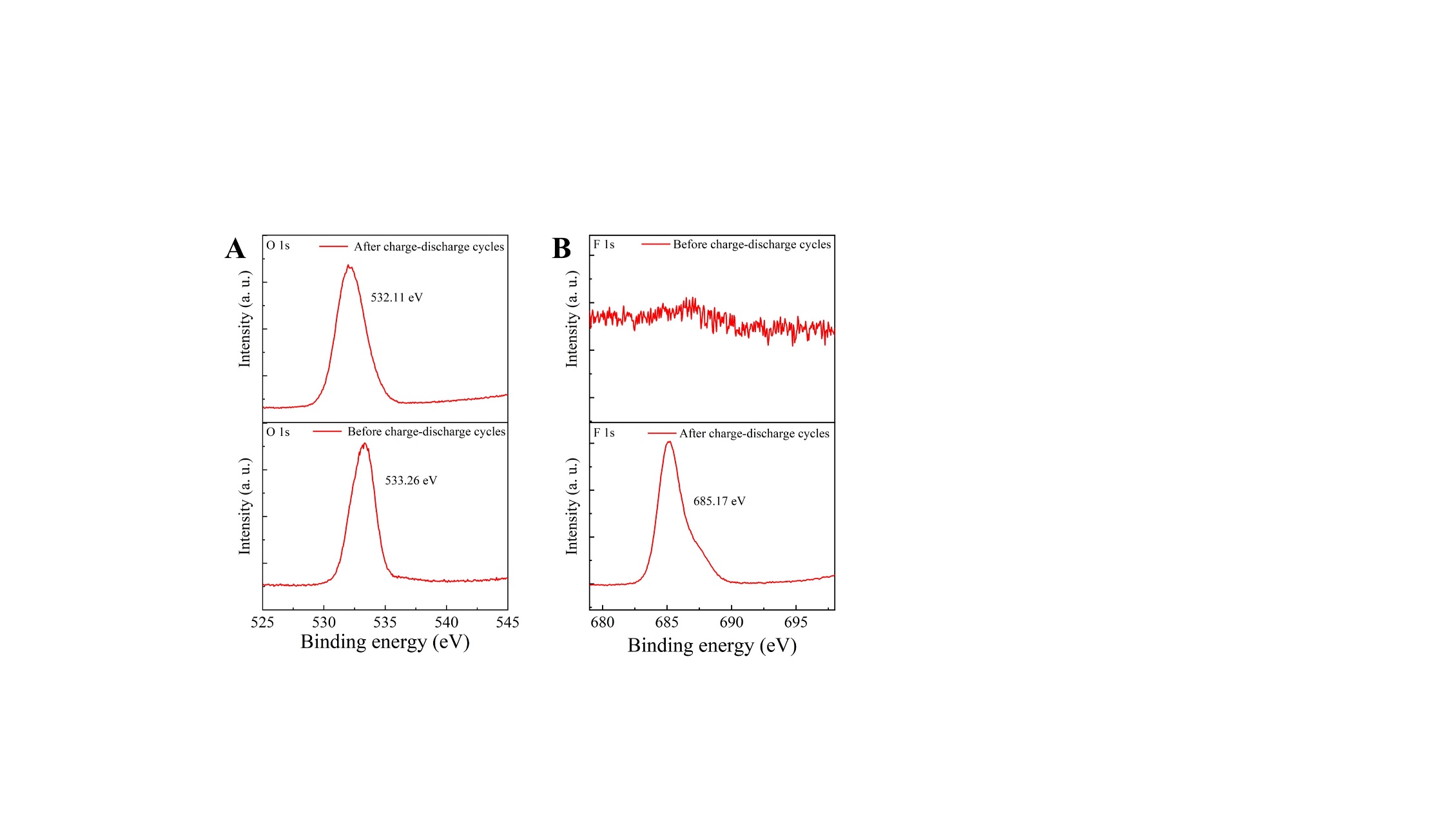
**

**S. 14. XPS analysis of silicon-carbon composite before and after cycling. (A) O 1s spectrum of silicon-carbon composite. (B) F 1s spectrum of silicon-carbon composite.**

**
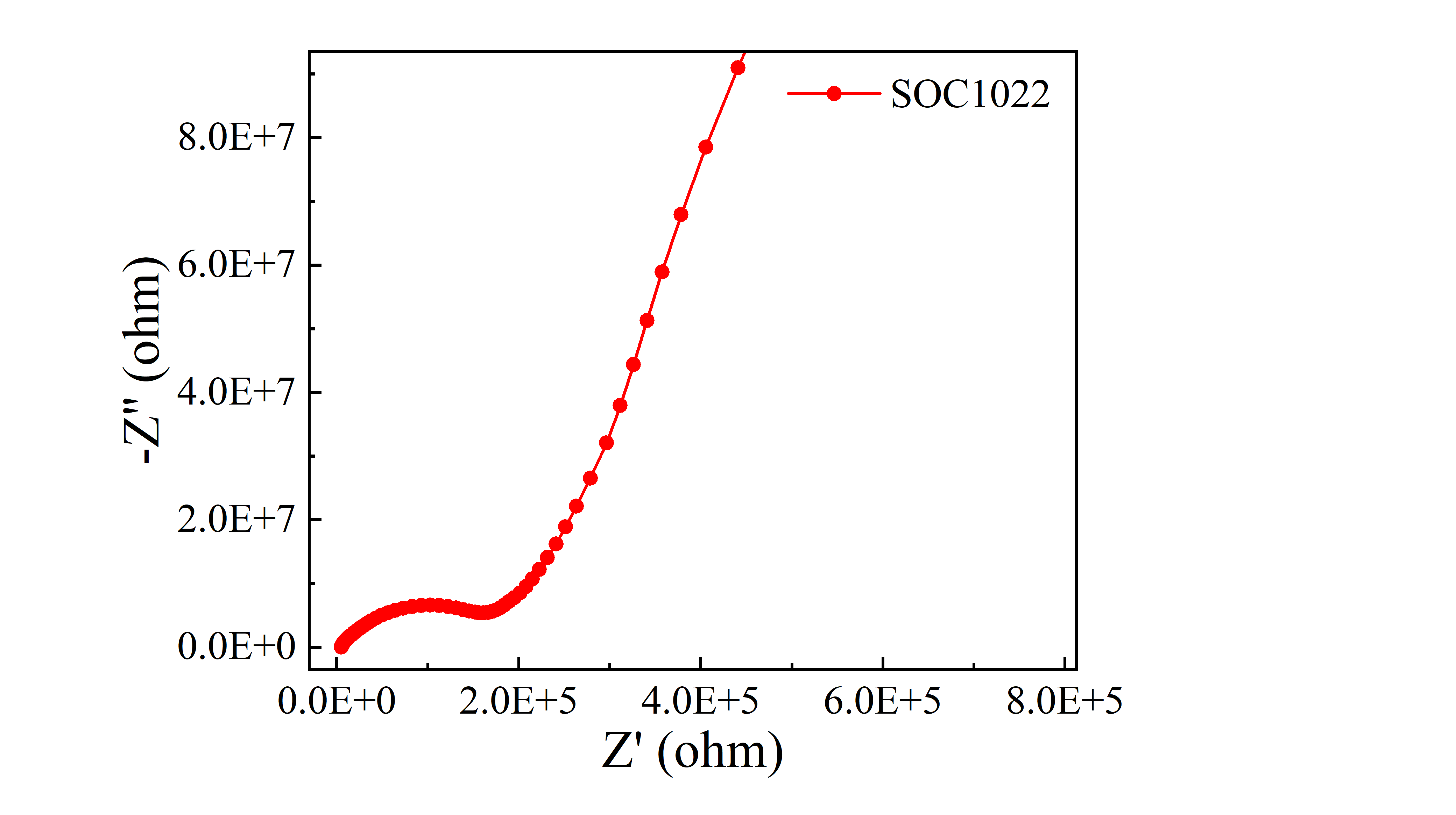
**

**S. 15. The electrochemical impedance spectroscopy measurement.**

**2. Training results without feature crosses.**

We supplemented the training results of the model without feature crosses layer. The loss function curve for 600 iterations is shown in the S.16A. As shown in S.16B, the predicted values for the initial discharge specific capacity can also exhibit a high degree of correlation with the true experimental values. The distribution of prediction errors, presented in S.16C, indicates that the majority of errors are confined within ± 200 mAh g^-1^.

To evaluate the contribution of each composition parameter to the prediction of the initial Coulombic efficiency, we performed a feature importance analysis based on permutation sensitivity. This analysis needs to be performed on the original features before feature crosses. Specifically, after computing the baseline root-mean-square error (RMSE) on the test set, we iteratively permuted each individual feature column in the test data while keeping the others fixed. For each permutation, predictions were made using the trained neural network, and the resulting RMSE was recalculated after inverse transformation. The importance of a feature was quantified as the increase in RMSE relative to the original value. A larger increase indicates a stronger influence of that feature on the model’s prediction accuracy. This method provides an intuitive, model-agnostic measure of feature importance by disrupting the relationship between individual input variables and the target output, thereby quantifying the extent to which model performance deteriorates in the absence of specific information. As shown in S.16D, the results are visualized as a horizontal bar chart, where each bar represents the increase in RMSE caused by permuting a particular compositional feature. Specifically, features 1-5 correspond to the concentrations of active material, carbon black, carbon additive, PVDF binder, and the type of carbon additive, respectively. The results indicate that the type of carbon additive exerts the greatest impact on enhancing the initial Coulombic efficiency. The prediction results for the initial Coulombic efficiency are presented in S.16E, while S.16F shows a sample-wise comparison between the predicted and true values. In the absence of feature crossover, GA predicts that the maximum initial discharge specific capacity is 5870.58 mAh g^-1^, even lower than the maximum value measured experimentally. It is much lower than the case where a feature crosses layer is added, which shows that feature crosses greatly improve the prediction performance by enhancing the correlation between features.


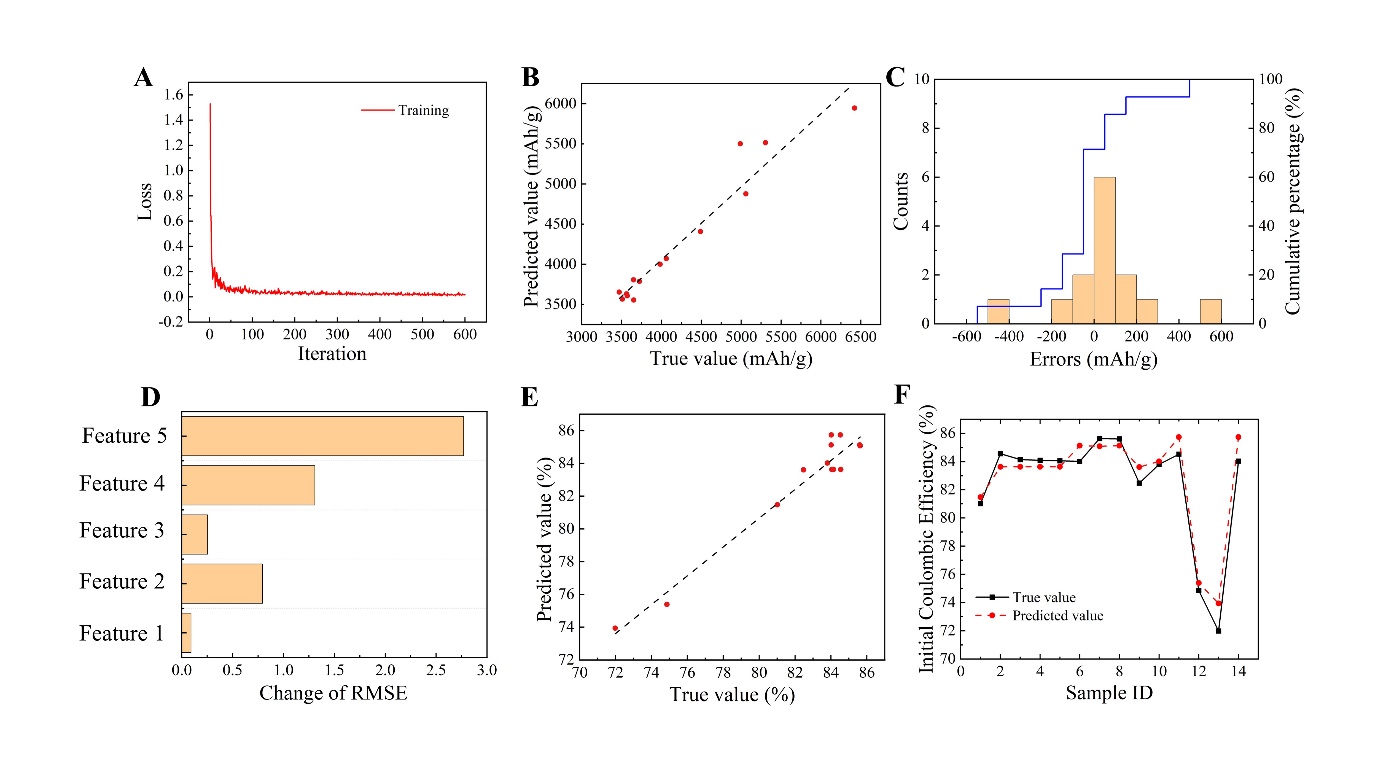


**S. 16. Training results without feature crosses.** (A) Loss function curve during training. (B) Comparison between predicted and true values of the initial discharge specific capacity. (C) Error distribution for the prediction of initial discharge specific capacity. (D) Feature importance analysis for the prediction of initial Coulombic efficiency. (E) Comparison between predicted and true values of the initial Coulombic efficiency. (F) Error analysis for the prediction of initial Coulombic efficiency.
